# Simultaneous Achievement of Enhanced Nonlinear Optical Absorption and Nonlinear Refraction in Highly Crystalline 2D Covalent Organic Frameworks Ultrathin Films

**DOI:** 10.1002/advs.202416170

**Published:** 2025-02-07

**Authors:** Tianyang Dong, Xingyuan Wen, Junyi Li, Xingzhi Wu, Chong Wang, Wenfa Zhou, Lingmin Yu, Yinglin Song, Chunru Wang, Li Jiang, Chunli Bai

**Affiliations:** ^1^ Beijing National Laboratory for Molecular Sciences Key Laboratory of Molecular Nanostructure and Nanotechnology Institute of Chemistry Chinese Academy of Sciences Beijing 100190 China; ^2^ University of Chinese Academy of Sciences Beijing 100049 China; ^3^ School of Physical Science and Technology Soochow University Suzhou 215123 China; ^4^ School of Physical Science and Technology Suzhou University of Science and Technology Suzhou 215009 China; ^5^ School of Materials and Chemical Engineering Xi'an Technological University Xi'an Shaanxi 710021 China; ^6^ School of Physics Harbin Institute of Technology Harbin 150001 China

**Keywords:** 2D COF, crystalline ultrathin films third‐order nonlinear optics, nonlinear optical absorption, nonlinear refraction

## Abstract

Large enhancement of nonlinear absorption and nonlinear refraction are achieved simultaneously in highly ordered two dimensional (2D) covalent organic framework (COF) films prepared by solidliquid interface one‐step method to overcome the weakness of COF powders in solubility. In the intrinsic nonlinear optical response obtained at 532 nm with 5 ns pulse, the nonlinear absorption coefficients (β) of two COF films are −4.87 × 10^−5^ and −1.29 × 10^−5^ m W^−1^, respectively. Simultaneously, the fitted closed‐aperture curves also show large nonlinear refractive indexes (*n*_2_), −5.62 × 10^−12^ m^2^ W^−1^ and −0.76 × 10^−12^ m^2^ W^−1^. The 4f coherent imaging performed at the same condition with a single‐shot pulse further verifies the outstanding nonlinear optical response without any damage probably experienced in the Z‐scan technique. Moreover, the differences in framework electronic structure and photoexcited states between two COF films are compared to explain the difference in nonlinear optical response. All the results indicate that two COF crystalline films with intrinsic giant nonlinear optical response can be capable of modulating both amplitude and phase of light, providing huge potential in all‐optical manipulating and switching at the nanoscale as outstanding nonlinear optical materials.

## Graphical abstract

Large enhancement of nonlinear absorption and nonlinear refraction are achieved simultaneously in highly ordered two dimensional (2D) covalent organic framework (COF) films prepared by solidliquid interface one‐step method to overcome the weakness of COF powders in solubility. The differences in framework electronic structure and photoexcited states are compared to explain the difference in nonlinear optical response in detail.

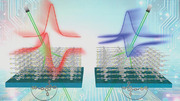

## 1 Introduction

Nonlinear optics (NLO) materials have been widely used in a number of advanced optical technologies including optical communications, optical switching, signal processing, photonic devices and laser protection, and so on since the invention of the laser in the 1960s.^[^ ^1^ ^]^ As one important response of NLO properties, third‐order NLO responses usually were concerned in the virtue of unique nonlinear absorption and nonlinear refraction properties. In detail, the nonlinear reverse saturation absorption (RSA) is useful for pulse shaping, mode locking, and optical limiting,^[^ ^2^ ^]^ the nonlinear saturation absorption (SA) has important applications in high‐density optical storage and optical information processing. The nonlinear refractive effect, in which the refractive index of the materials experienced obvious change when exposed to intense light, can be used for optical switches and optical modulators.^[^ ^3^ ^]^ A material both with strong nonlinear absorption and strong nonlinear refraction always is the ultimate goal to pursuit. Regrettably, most organic NLO materials only display a single advantage either in nonlinear absorption or in nonlinear refraction up to now.

2D layered materials have attracted extensive research attention in the past decades because of their unique electrical properties and their fascinating optical, mechanical, thermal, and chemical properties.^[^ ^4^ ^]^ The research on NLO properties of 2D materials began with graphene^[^ ^1^, ^5^ ^]^ followed by newly emerging 2D materials, such as transition metal dichalcogenides (TMDs),^[^ ^3^, ^6^ ^]^ Black phosphorus (BP),^[^ ^3^ ^]^ Hexagonal boron nitride (*h*‐BN),^[^ ^7^ ^]^ MXenes,^[^ ^8^ ^]^ 2D perovskite materials^[^ ^9^ ^]^ and Metal Organic Frameworks.^[^ ^10^ ^]^ These 2D layered inorganic nanomaterials generally show ultrafast broadband optical response, large optical nonlinearities absorption, and strong excitonic effects.^[^ ^11^, ^12^ ^]^ Meanwhile, these 2D materials leave much to be desired in stability and diversity for practical application.^[^ ^13^ ^]^ As an emerging class of novel 2D materials in the past decade, crystalline 2D covalent organic frameworks (COFs) with extremely diverse geometries and ultrahigh stability offer a powerful molecular platform for complex structural design and tailor‐made for various research areas such as gas storage, chemical separation, sensors, energy storage, and catalysis.^[^ ^14^ ^]^ Moreover, the long‐range orderly *π*‐conjugated framework structure with adjustable electron properties of 2D COFs also stimulated to explore for diverse optical applications including nonlinear optics.^[^ ^15^ ^]^

Since the third‐order NLO polarizability has been confirmed to benefit from the delocalization of the *π*‐electron cloud and charge transfer from donors to acceptors,^[^ ^16^ ^]^ 2D COFs with huge *π*‐conjugated backbone frameworks constructed by diverse donor units and acceptor units are anticipated to be ideally potential third‐order NLO materials. Feng et al. first studied the NLO properties of porphyrin COFs powders dispersed in isopropanol with different metal centers (Por‐COFs‐M_1_M_2_) and showed a large nonlinear absorption coefficient as high as 4500 cm GW^−1^.^[^ ^17^ ^]^ Deng et al. investigated the TPA properties of six 2D COFs crystals with different linking chromophores and proved that the ordered arrangement of molecular chromophores to be a key chemical approach of achieving high two‐photon absorption (TPA) performance of materials.^[^ ^18^ ^]^ The same positive effects of the highly ordered lattice structure in ether‐linked porphyrin COFs dispersed in DMF on NLO properties were verified by Chen et al. from visible to near‐infrared wavelength.^[^ ^11a^ ^]^ Xie et al. found that the NLO properties of 2D porphyrin‐based COFs are a little better than 3D porphyrin COFs when dispersed in an alcohol solution.^[^ ^19^ ^]^ Free‐standing COF films appeared to overcome the insoluble powder unsuited to perform NLO measurements to some extent. Wong et al. obtained prominent wideband nonlinear saturable absorption (SA) in a kind of free‐standing COF films with controllable thickness and enhanced *π*‐conjugation.^[^ ^20^ ^]^ Biswal et al. then reported a cyclotriphosphazene core with vinylene‐linked COF self‐standing film with a reverse saturable absorption coefficient of 58.37 cm·GW^−1^.^[^ ^21^ ^]^ Jia et al. successfully prepared uniform donor–acceptor (D–A) COFs films to present a large nonlinear absorption coefficient (*β*) of 1.83 × 10^6^ cm·GW^−1^, far better than most reported third‐order NLO materials.^[^ ^22^ ^]^ Then, they reported another phenothiazine‐based COF (TFP‐TZ) film using the same method to obtain simultaneously a *β* value of −6.78 × 10^−7^ m·W^−1^ and an outstanding nonlinear refraction coefficient (*n*_2_) of −1.67 × 10^−13^ m^2^ W^−1^.^[^ ^23^ ^]^ These outstanding results encouraged us to explore more NLO materials based on 2D COFs. Regrettably, the extremely poor solubility of 2D COFs powders seriously limit their deep development as NLO materials. Pursuing directly the highly crystalline films of 2D COFs with reasonable structure design is expected to be an effective path to achieve both excellent nonlinear absorption and excellent nonlinear refraction.

In this context, 1,3,6,8‐tetrakis(4‐aminophenyl) pyrene and [3,2‐B]thiophene‐2,5‐dicarboxaldehyde, 2,2′‐bithiophene‐5,5′‐dicarboxaldehyde were selected to construct Py‐TT COF and Py‐BT COF, respectively. Pyrene‐ and thiophene‐based derivatives have been verified to be outstanding NLO chromophores in previous studies.^[^ ^24^ ^]^ Py‐TT and Py‐BT COF powders were first reported for proton conductivity by Wang et al.^[^ ^25^ ^]^ In this article, Py‐TT and Py‐BT COFs were prepared in the form of highly crystalline films by using the solid‐liquid interface method^[^ ^26^ ^]^ to be used directly as NLO devices, in which imine linkage was chosen to achieve better crystallization for its strong self‐repairing ability. The NLO properties of Py‐TT and Py‐BT films were investigated both by Z‐scan technique and 4*f* coherent imaging. Both two results show that the two 2D COFs not only possess outstanding nonlinear saturated absorption but also exceptional nonlinear refraction. The nonlinear absorption coefficient (*β*) of Py‐TT films is −4.87 × 10^−5^ m W^−1^ and that of Py‐BT films is −1.29 × 10^−5^ m W^−1^, respectively. The nonlinear refractive index (*n*_2_) of Py‐TT film is −5.62 × 10^−12^ m^2^ W^−1^ and that of Py‐BT film is −0.76 × 10^−12^ m^2^ W^−1^, respectively. The results show that crystalline films of Py‐TT and Py‐BT COF are a kind of outstanding NLO materials, moreover, the condensed thiophene ring structure in Py‐TT can improve further NLO properties compared with Py‐BT based on 2,2′‐bithiophene. The transient photocurrent response, surface photovoltage spectra (SPV), and time‐resolved photoluminescence were utilized to understand successfully the photoexcited states of COF films and elucidate the underlying principles between NLO properties and COFs structures.

## 2 Results and Discussion

**Py‐TT COF film**: 1,3,6,8‐tetrakis(4‐aminophenyl)pyrene (0.032 mmol, 18 mg) and Thieno[3,2‐b]thiophene‐2,5‐dicarboxaldehyde (0.063 mmol, 12.5 mg) were added to a mixture of mesitylene/benzyl alcohol in a 15 mL sealed pressure‐resistant reaction tube (PRRT). An orange suspension was obtained after sonification. Then 0.5 mL of 3 m acetic acid was added. A quartz sheet (1.5 cm × 4.7 cm) was inserted into the PRRT and immersed in the precursor solution. The pressure‐resistant glass reaction tube is subjected to the traditional three‐cycle method of liquid nitrogen freezing, degassing, and thawing degassing. After that, place the sealed PRRT in an oven at 120 °C for 4 days. After cooling to room temperature, the obtained quartz sheet was immersed in dry MeCN and dried with compressed air.

**Py‐BT COF film**: 1,3,6,8‐tetrakis(4‐aminophenyl)pyrene (0.028 mmol, 16 mg) and 2,2′‐bithiophene‐5,5′‐dicarboxaldehyde (0.056 mmol, 12.5 mg) were added to a mixture of mesitylene/benzyl alcohol in a 15 mL sealed PRRT. An orange suspension was obtained after sonification. Then 0.5 mL of 3 m acetic acid was added. The quartz sheet (1.5 cm × 4.7 cm) was inserted into the PRRT and immersed in the precursor solution. The pressure‐resistant glass reaction tube is subjected to the traditional three‐cycle method of liquid nitrogen freezing, degassing, and thawing degassing. After that, place the sealed PRRT in an oven at 120 °C for 4 days. After cooling to room temperature, the obtained quartz sheet was immersed in dry MeCN and dried with compressed air.

**Figure 1:**
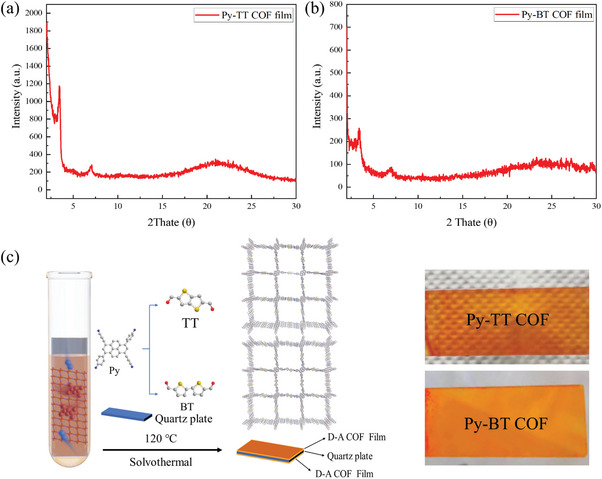
The Swept‐angle XRD of a) Py‐TT COF film and b) Py‐BT COF film; c) Schematic diagram of synthesis scheme and structure of Py‐TT COF films and Py‐BT COF films.

Due to Py‐TT and Py‐BT COF powders have been ever reported in the previous literature, most of the structure characterizations on powders are listed in the supporting information. Powder X‐ray diffraction (PXRD, Figures S1 and S2, Supporting Information) confirmed that Py‐TT and Py‐BT have fine crystallinity without any visible amorphous background. The COF films were first tested by grazing angle XRD at 0.5° incidence angle and an intense diffraction peak at 2*θ* = 3.2° was found to verify the crystallinity of the films (**Figure** **1**). The strong envelope peak at 21° is attributed to quartz substrates. In the Fourier transform infrared (FTIR) spectroscopy, the typical C═N vibration band at 1620 cm^−1^ appears both in the spectra of Py‐TT and Py‐BT films, revealing the formation of imine linkages (Figure S3, Supporting Information). The ^13^C cross‐polarization magic‐angle spinning (CP‐MAS) NMR spectroscopy (Figures S5 and S6, Supporting Information) presented a chemical shift of 148 ppm to confirm the formation of imine bonds both for Py‐TT and Py‐BT.^[^ ^27^, ^28^ ^]^

**Figure 2:**
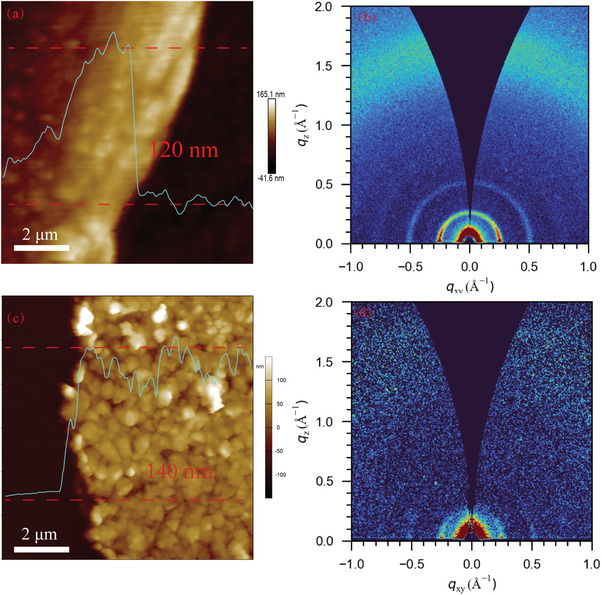
AFM image of a) Py‐TT COF film and c) Py‐BT COF film on a quartz plate, with the height profile determined; GIWAXS scattering patterns of b) Py‐TT COF film and d) Py‐BT COF film.

Atomic force microscopy (AFM) was performed to investigate the morphology and measure the film thickness of the two COF films. As shown in **Figure** **2**a,b, both Py‐TT and Py‐BT films display good densification with uniform surfaces in keeping with the photos shown in Figure 1c. But the obvious difference existed in film thickness can be seen in the inset curves of AFM images. The thickness of Py‐TT film is ≈120 nm and that of Py‐BT film is ≈140 nm. The arithmetic average deviation (*R*_a_) and root mean square deviation of the contour (*R*_q_) are utilized to evaluate the roughness of thin‐film materials. For Py‐TT COF, the *R*_a_ and *R*_q_ values are 51.3 and 35.9 nm. For Py‐BT COF, however, the *R*_a_ and *R*_q_ values are 66.7 and 49.6 nm, respectively. The grazing‐incidence wide‐angle X‐ray scattering (GIWAXS) is performed to investigate the molecular orientation in the COF films, which is paramount for their optical properties. From the similar diffraction patterns of two COF films shown in Figure 2c,d. Intense signals appear in the in‐of‐plane direction to prove the parallel orientation of COF films to the quartz surface. Moreover, both two COF films have fine crystallinity according to the data of the 2D synchrotron radiation GIWAXS experiment listed in Figure S11 (Supporting Information), in which the main oriented crystal plane corresponds to the (110) crystal plane of the XRD.

**Figure 3:**
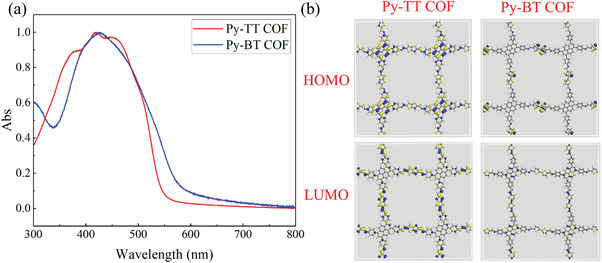
a) UV–vis spectra of Py‐TT COF film and Py‐BT COF film on quartz plate; b) Molecular frontier orbitals calculated for Py‐TT COF and Py‐BT COF by Dmol3 module of Material Studio 2020.

From similar UV–vis absorption curves shown in **Figure** **3**, the optical bandgap of Py‐TT COF and Py‐BT COF can be estimated to be 2.23 and 2.08 eV, respectively. The density functional theory (DFT) calculations were performed to further determine their HOMO and LUMO using a Dmol3 module of Material Studio 2020, in which the gap of Py‐TT and Py‐BT were calculated to 1.79 and 1.32 eV, respectively. It is found that the electron density of HOMO in Py‐TT is predominantly located on pyrene units, as shown in Figure 3b, which means pyrene is the electron donor. In contrast, the opposite electron distribution was found in Py‐BT with pyrene units being the electron acceptors and bithiophene units as electron donors.

In order to understand the difference in band structure, the work function (WF) values of the COF films were determined using Kelvin probe force microscopy (KPFM). The WF of Py‐TT and Py‐BT are determined to be 5.03 and 5.10 eV, respectively. The CPD values of Py‐TT and Py‐BT films with platinum probe were −270.6 and −197.4 mV as shown in Figures S9 and S10 (Supporting Information), respectively. The CPD value of Py‐TT obviously was ≈73 mV lower than that of Py‐BT, suggesting superior donor–acceptor (D–A) regulation in Py‐TT COF. It is believed that the different electronic structure induced by the structural modifications would eventually influence their nonlinear optical properties.

**Figure 4:**
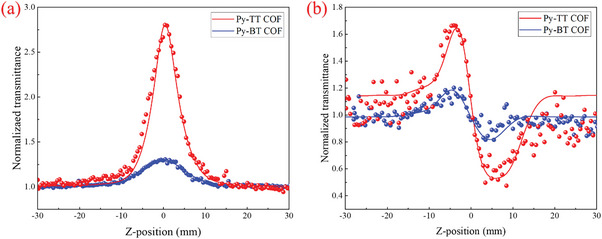
a) The open‐aperture Z‐scan curves of Py‐TT COF and Py‐BT COF; b) The close‐aperture Z‐scan measurements of Py‐TT COF and Py‐BT COF.

The third‐order NLO properties of two COF films were examined directly via open‐ and closed‐aperture Z‐scan techniques at 532 nm with 5 ns laser pulses.^[^ ^29^ ^]^ The results of the open‐aperture Z‐scan curves showed that the normalized transmittance of Py‐TT and Py‐BT films gradually increased as the sample approached focus, exhibiting a saturable absorption (SA) response at a pulse energy of 15 *µ*J. At the beam focus, the normalized transmittance of the Py‐TT and Py‐BT films are 2.38 and 1.35, respectively (**Figure** **4**a). The linear transmittance of the Py‐TT and Py‐BT films at 15 *µ*J are 29% and 15%, respectively. Furthermore, the nonlinear absorption coefficients (*β*) of the Py‐TT and Py‐BT films under the above condition are extracted from numerical simulation (see ESI†) as −4.87 × 10^−5^ and −1.29 × 10^−5^ m·W^−1^ for Py‐TT and Py‐BT, respectively. Both two COF films display much larger nonlinear absorption coefficients than the previous reported COFs’ powders dispersed in solution. It is believed that highly ordered crystalline COF films can enhance greatly the NLO response. Moreover, the *β* value of Py‐TT is two times of Py‐BT at the same condition. The stronger intramolecular electron transfer in Py‐TT constructed by rich electron fused‐ring thiophene units, which was verified by the lower bandgap, should be responsible for the larger *β* value.

The obvious peak‐valley mode in closed‐aperture curves of Z‐scan measurement is shown in Figure 4b. In the two closed‐aperture curves, the normalized linear transmittances experienced first increasing and then decreasing to present peak‐valley shapes when the films moved from −Z via 0 to +Z. The curve shape means the two COF films all possess a self‐defocusing effect resulted from negative nonlinear refractions (*n*_2_). The difference between the peak and valley was found to be 1.2 and 0.4 for Py‐TT and Py‐BT, respectively. According to numerical simulation (see ESI†), the *n*_2_ of Py‐TT and Py‐BT films are calculated to be −5.62 × 10^−12^ and −0.76 × 10^−12^ m^2^·W^−1^, respectively. As far as we know, there is no nonlinear refraction effect ever reported in the dispersed solution of COF powders, these two *n*_2_ values are the highest nonlinear refraction coefficients in organic NLO materials. More, *n*_2_ of Py‐TT film has larger nonlinear refraction than that of Py‐BT film, which is still believed to result from stronger intramolecular electron transfer between pyrene units and rich electron fused‐ring thiophene units, similar to the nonlinear absorption case above. It should be pointed out that the self‐defocusing curve of Py‐TT film lacks perfect symmetry with suppressed peak and deepened valley, which is apparently the influence of giant SA in Py‐TT.

The third‐order nonlinear polarization coefficient (χ^(3)^) values are further calculated according to the following equation based on their *β* values and *n*_2_ values to evaluate the performance of third‐order NLO:

(1) $$Re\left\{x^{\left(3\right)}\right\}=\frac{n_{0}^{2}c}{120\pi^{2}}n_{2}$$

(2) $$Im\left\{x^{\left(3\right)}\right\}=\frac{n_{0}^{2}c^{2}}{240\pi^{2}\omega }\beta_{eff}$$

(3) $$\left|x^{\left(3\right)}\right|=\left|Re\left\{x^{\left(3\right)}\right\}+Im\left\{x^{\left(3\right)}\right\}\right|$$

where *c* is the velocity of the light in vacuum, *n*_0_ is the linear refractive index, and *ω* is the angular frequency of the excitation wavelength.^[^ ^30^ ^]^ In results, the *χ* ^(3)^ of Py‐TT is calculated to be −41.50 × 10^−7^ *esu*, which is 7 times of Py‐BT (−5.84 × 10^−7^ *esu*). All the above obtained third‐order NLO parameters of Py‐BT and Py‐TT COF films are summarized in **Table** **1** for convenient comparison.

**Table 1:** The third‐order NLO parameters of Py‐TT COF and Py‐BT COF.

Film Samples	Wavelength, pulse	Z‐scan measurement			4*f* coherent	
		*β* [10^−5 ^m W^−1^]	*n*_2_ [10^−12^ m^2^ W^−1^]	*χ* ^3^ [10^−7^ esu]	*β* [10^−5 ^m W^−1^]	*n*_2_ [10^−12^ m^2^ W^−1^]
**Py‐TT**	532 nm, 5ns	−4.87	−5.62	−41.50	−2.96	−3.77
**Py‐BT**	532 nm, 5ns	−1.29	−0.76	−5.84	−2.92	−0.93

Considering that COF films could be easily damaged when exposed continuously under a pulsed laser in the Z‐scan technique so that the non‐intrinsic nonlinear optical responses could dominate Z‐scan results. Therefore, single‐shot 4*f* coherent imaging technology was used on the two COF films to eliminate the unwanted damage to obtain a more accurate and intrinsic NLO response with higher sensitivity. The 4*f* imaging system is based on the Zernike spatial filtering principle to transform the phase changes caused by non‐linear refraction into intensity changes in the image plane.^[^ ^31^ ^]^ Three kinds of imaging data are needed in the experiment, i.e., no sample image, linear image, and nonlinear image. No sample image acquisition is done without no sample in the system by placing high‐density neutral filters before the charge‐coupled device (CCD). The linear and nonlinear images are acquired by placing the same amount of neutral filters before and after the samples, respectively. The linear and nonlinear images are then used to calculate the nonlinear absorption coefficient and refraction index n_2_. Based on G. Boudebs's theory of 4*f* imaging^40^, the nonlinear absorption coefficient and refraction index can be extracted through numerical simulation.

The transmitted energy can be acquired by integrating all the pixels of each image. The transmittance change of the sample can be obtained by dividing the intensity of the nonlinear image by the intensity of the linear image to get the nonlinear absorption coefficient. The nonlinear signal processing leads to *ΔT* using the difference between the mean intensity inside and outside of the phase object (central) region to get a nonlinear refraction index. Only nonlinear images are provided in the text with the left images being listed in ESI† for convenient reading.

**Figure 5:**
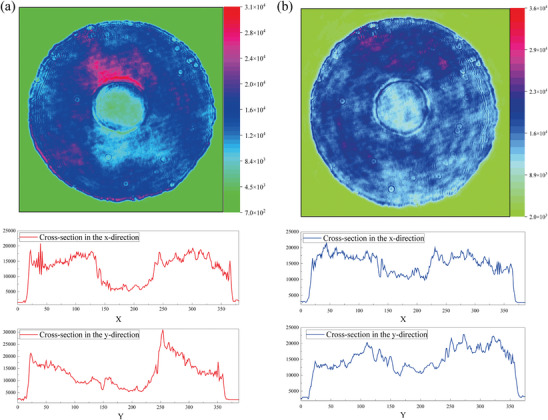
The nonlinear images of a) Py‐TT COF and b) Py‐BT COF obtained by experiment, central area means PO area, X cross‐section (up), Y cross‐section (under).

In the obtained **Figure** **5** at 532 nm, 5 ns, and 6.8 *u*j display a much weaker central region in Py‐TT, forming an obvious concave curve shape. A similar phenomenon could also be found in Py‐BT but with less contrast between the central and donut‐like regions. The concave center in Figure 5 indicated that both two COF films show a negative nonlinear refractive index, which agrees with the Z‐scan results. The numerical simulation gives fitting results of nonlinear refractive coefficients as −3.77 × 10^−12^ m^2^·W^−1^ for Py‐TT and −9.32 × 10^−13^ m^2^·W^−1^ for Py‐BT, respectively. The results, further confirm the intrinsic giant negative nonlinear refraction response in both COF films. The results suggest Py‐TT and Py‐BT COF films are capable to modulate both amplitude and phase of light, providing huge potential in all‐optical manipulating and switching at the nanoscale as outstanding nonlinear optical materials.

**Figure 6:**
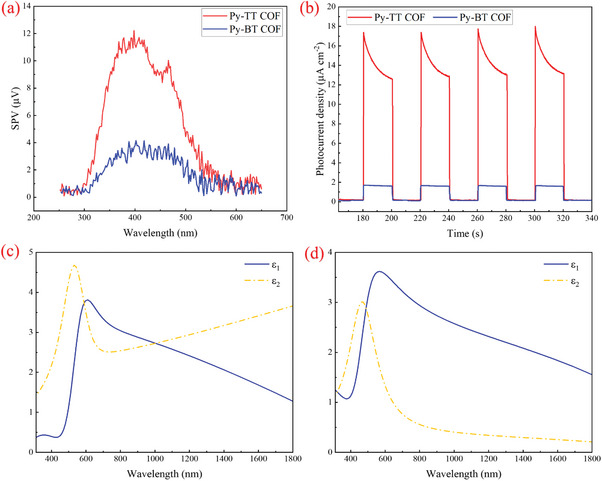
a) The SPV spectra of Py‐TT COF films and Py‐BT COF films; b) Transient photocurrent responses of Py‐TT COF films and Py‐BT COF films; real (blue solid curve) and imaginary parts (yellow dashed curve) of the permittivity of c) Py‐TT COF and d) Py‐BT COF films.

The nonlinear optical responses are influenced by various factors, among which the photo‐induced carrier dynamics are believed to play a very significant role.^[^ ^32^ ^]^ To deeply understand the difference in NLO response between Py‐TT and Py‐BT, the carrier dynamics were investigated through transient photocurrent response and surface photovoltage spectroscopy (SPV) (**Figure** **6**; Figures S15 and S16, Supporting Information). The transient photocurrent response shown in Figure 6a confirms the efficient and rapid generation and separation of photo‐induced charges in these two COF films.^[^ ^33^ ^]^ At 0 V voltage, Py‐TT films generate a photocurrent of 27.81 mA·cm^−^^2^, which is ≈40 times that of Py‐BT (0.68 mA·cm^−^^2^). While the SPV signal of Py‐TT film is stronger 4 times than that of Py‐BT, indicating a more effective separation of charge carriers in Py‐TT in Figure 6b.^[^ ^34^ ^]^ The internal electric fields (IEF) in Py‐TT and Py‐BT were calculated using the following Equation (4) developed by Kanata et al.^[^ ^35^ ^]^

(4) $$F_{s}=\sqrt{-2V_{s}\rho /\varepsilon \varepsilon_{0}}$$

in which, *F*s represents the magnitude of IEF, *V*s represents the surface voltage, *ρ* represents the surface charge density, *ε* represents the low‐frequency dielectric constant, and *ε*_0_ denotes the permittivity of free space. The magnitude of IEF is mainly determined by the surface voltage (*V*s) and charge density (*ρ*) since both *ε* and *ε*_0_ remain constant for a given sample. The 12 times of IEF intensity in Py‐TT compared with Py‐BT indicates more efficient charge carriers’ mobility.^[^ ^34^, ^36^ ^]^ From the equation, it is known that IEF can be enhanced by the low dielectric constant *ε* of the sample.^[^ ^37^ ^]^ Consequently, the dielectric characteristics (*ε*) of Py‐TT and Py‐BT films were investigated using ellipsometry (Figure 6c,d). From the real parts of permittivity shown in blue solid curves, it can be seen that *ε* of Py‐TT is much smaller than that of Py‐BT at 532 nm.

**Figure 7:**
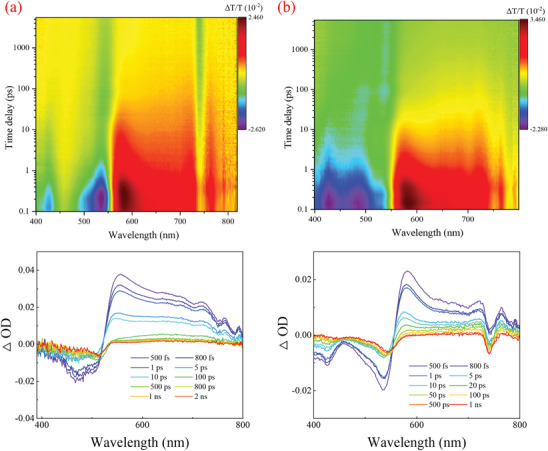
fs‐TA spectra of a) Py‐TT film and b) Py‐BT film.

Femtosecond transient absorption (fs‐TA) spectroscopy was utilized to investigate the ultrafast charge transfer dynamics of COF films. The obtained spectra are displayed in **Figure** **7**. Analysis of the fs‐TA spectra employing the global analysis method enables the extraction of evolution‐associated spectra (EAS). Figure 7a revealed that the spectra of Py‐TT experienced a transition from a negative to a positive signal in the range of 390–800 nm. The negative signal ranging from 390 to 520 nm can be attributed to ground‐state bleach (GSB), which is supported by UV absorption peaks of Py‐TT COF in the same wavelength range in UV–vis spectra. A positive signal is observed from 520 to 800 nm, which arises from the competition between reverse and forward saturable absorption processes. Within the time range of 0.5–2000 ps, the negative signals persist and undergo continuous evolution, suggesting the generation of long‐lived ground‐state bleach (GSB). This phenomenon can be attributed to the decay of the triplet state of Py‐TT COF nanofilms to the ground state. The kinetics of Py‐BT COF are identical to those of Py‐TT COF, with the negative signal at 780 nm attributed to the laser signal.

Global analysis gave the identification of three main components with lifetimes of 0.8, 30, and 3930 ps. The remarkably short lifetime of 0.8 ps likely corresponds to the formation of excited states associated with local excitation states (LES). The lifetime of 30 ps corresponds to the transition from the singlet state to the triplet state through a process known as intersystem crossing (ISC). The longest lifetime, measured at 3930 ps, corresponds to the lifetime of triplet states. These lifetimes are consistent with the previously reported femtosecond fluorescence lifetimes of 13 ps, wherein the decay from the singlet state to the ground state predominantly takes place via non‐radiative pathways. The electronic relaxation process can be summarized as follows: upon laser irradiation, Py‐TT films undergo a rapid transition to the S_n_ state, followed by internal conversion to the S_1_ state. Subsequently, the electrons at the S_1_ state underwent intersystem crossing to the triplet state instead of promptly return to the ground state. The dynamic process of Py‐BT is identical to that of Py‐TT, only with different lifetimes: 0.8, 22.8, and 1260 ps (Figure S18b, Supporting Information).

**Figure 8:**
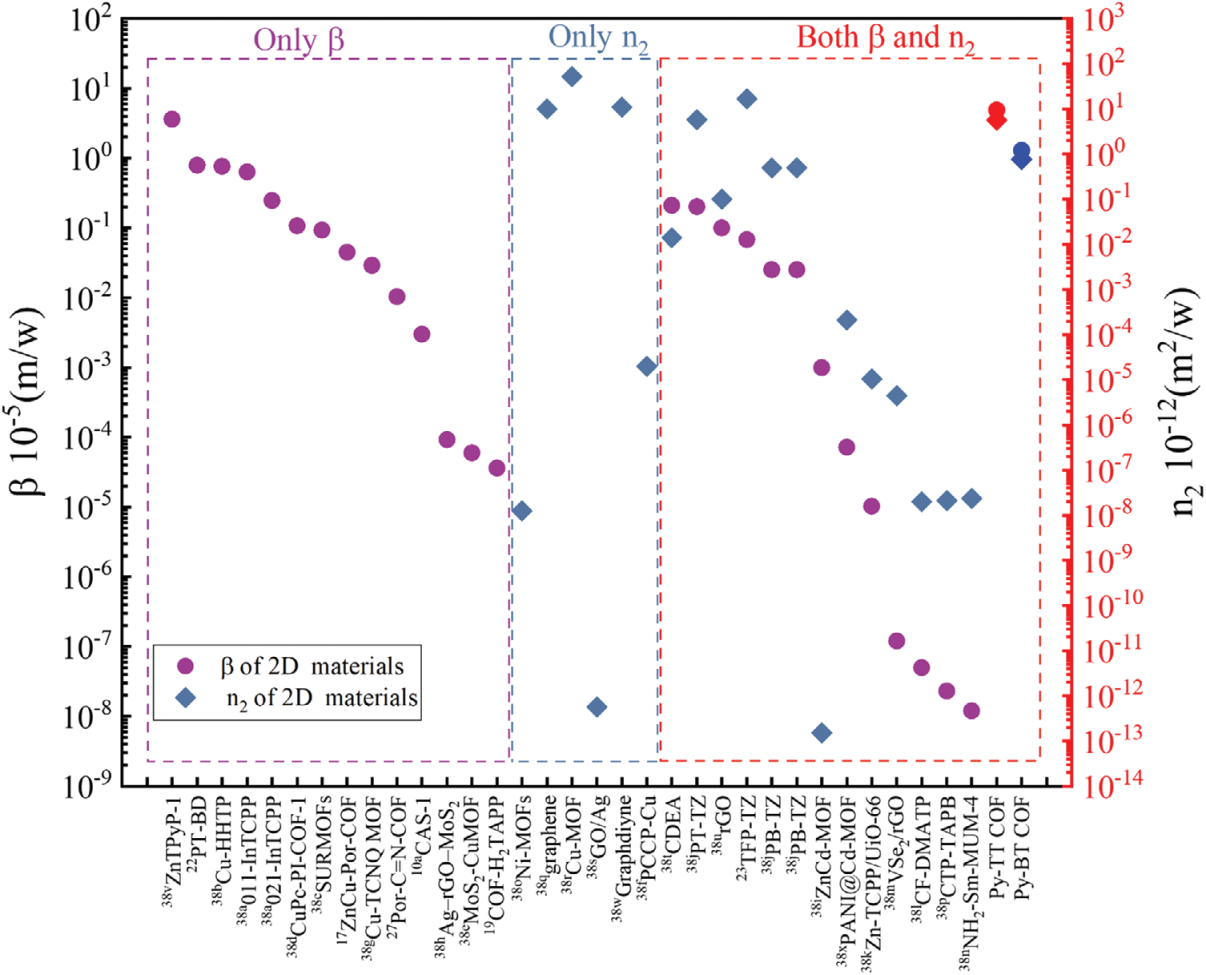
The comparison of 𝛽 and γ of the Py‐TT COF and Py‐BT COF with representative 2D organic materials at 532nm.^[^ ^10^, ^17^, ^19^, ^22^, ^23^, ^27^, ^38^ ^]^

The outstanding NLA and NLR coefficients of Py‐TT and Py‐BT COF films were listed in the graph of **Figure** **8** with other reported 2D organic materials for comparison. It is easy to see that the advantage of Py‐TT and Py‐BT COF films is not only in nonlinear absorption but also in nonlinear refraction.

## 3 Conclusion

In summary, we successfully synthesized two types of COF nanofilms on quartz substrates with highly crystalline orientation using the solid‐liquid interface method. The films exhibited high crystallinity in‐plane orientation as confirmed by GIWAXS. Py‐TT COF and Py‐BT COF are constructed by pyrene and bithiophene (Py‐BT) or thiophthene (Py‐TT) using imine as the linkage bridge between conjugated units in the plane. The obtained huge and ordered conjugation framework eventually presented enhanced nonlinear optical response with van der Waals forces and *π*–*π* stacking between planes. The Z‐scan results obtained at 532 nm with 5 ns pulse show that the nonlinear absorption coefficients (*β*) of Py‐TT and Py‐BT COF films are −4.87 × 10^−5^ and −1.29 × 10^−5^ m W^−1^, respectively. Simultaneously, the fitted closed‐aperture Z‐scan shows large nonlinear refractive indexes (*n*_2_), −5.62 × 10^−12^ m^2^ W^−1^ for Py‐TT and −0.76 × 10^−12^ m^2^ W^−1^ for Py‐BT COF films. The 4*f* coherent imaging performed at the same condition with a single pulse further verifies the outstanding nonlinear refractive responses without any possibility of thin film damage, which is probably experienced in the Z‐scan technique. Moreover, the differences in framework electronic structure and photoexcited states between Py‐TT and Py‐BT were compared to explain the better nonlinear optical response of the former than that of the latter. All the results indicate that Py‐TT and Py‐BT COF crystalline films with intrinsic giant nonlinear response can be capable of modulating both amplitude and phase of light, providing huge potential in all‐optical manipulating and switching at the nanoscale as outstanding nonlinear optical materials.

## Conflict of Interest

The authors declare no conflict of interest.

## Supporting information



Supporting Information

## Data Availability

The data that support the findings of this study are available in the supplementary material of this article.
