# Supplementary material for: Simultaneous Achievement of Enhanced Nonlinear Optical Absorption and Nonlinear Refraction in Highly Crystalline 2D Covalent Organic Frameworks Ultrathin Films
Source: Adv Sci (Weinh). 2025 Feb 7;12(13):2416170. doi: 10.1002/advs.202416170 (PMC11967766; doi:10.1002/advs.202416170)
Supplement: Supplementary file 1 — Supporting Information [file ADVS-12-2416170-s001.docx]

**Simultaneous Achievement of Enhanced Nonlinear Optical Absorption and Nonlinear Refraction in Highly Crystalline 2D Covalent Organic Frameworks Ultrathin Films**

*Tianyang Dong^1,2^, Xingyuan Wen^1^, Junyi Li^4^, Xingzhi Wu^5^, Chong Wang^1,2^, Wenfa Zhou^4^, Lingmin Yu^6^, Yinglin Song^3,4*^, Chunru Wang^1,2^, Li Jiang^1,2*^, ChunLi Bai^1,2*^*

*T. Y. Dong, C. wang, X.Y. Wen, Associate Prof. L. Jiang, Prof. C. R. Wang, Prof. C. L. Bai*

^1^*Beijing National Laboratory for Molecular Sciences, Key Laboratory of Molecular Nanostructure and Nanotechnology, Institute of Chemistry, Chinese Academy of Sciences, Beijing 100190, China.*

^2^*University of Chinese Academy of Sciences, Beijing 100049, China*

*E-mail: jiangli@iccas.ac.cn; clbai@cas.cn.*

Prof. Y. L. Song

*^3^School of Physics, Harbin Institute of Technology, Harbin 150001, China*

^4^*School of Physical Science and Technology, Soochow University, Suzhou 215123, China*

*E-mail: ylsong@hit.edu.cn*

W. F. Zhou, J. Y. Li

^4^*School of Physical Science and Technology, Soochow University, Suzhou 215123, China*

*E-mail: ylsong@hit.edu.cn*

Prof. X. Z. Wu

^5^*School of Physical Science and Technology, Suzhou University of Science and Technology*

Prof. L. M. Yu

^6^*School of Materials and Chemical Engineering, Xi’an Technological University, Xi’an, Shaanxi, 710021, China*

**Materials and synthesis**

**1. Materials**

All solvents and reagents were used as received without further purification unless otherwise specified. 1,3,6,8-tetrakis(4-aminophenyl) pyrene (purity ≥ 98%) was purchased from macklin Co.

[3,2-b] thiophene-2,5-dicarboxaldehyde (purity ≥ 98%) and 2,2'-bithiophene-5,5'-dicarboxaldehyde (purity ≥ 98%) were purchased from J&K Scientific Co. Benzyl alcohol (purity ≥ 99.5%) and 1,3,5-trimethylbenzene (purity ≥ 98%) were purchased from J&K Scientific Co. Acetic acid (purity ≥ 99.5%) was purchased from tcichemicals Co. 1,4-dioxane (purity ≥ 99.8%) was purchased from J&K Scientific Co. Tetrahydrofuran (purity ≥ 99.8%) was purchased from Concord Technology Co.

**2. The synthesis of covalent organic frameworks**

**Py-TT COF powder:** 1,3,6,8-tetrakis(4-aminophenyl)pyrene (0.064 mmol, 36 mg) and thieno[3,2

b]thiophene-2,5-dicarboxaldehyde (0.127 mmol, 25 mg) were added to a mixture of mesitylene/benzyl alcohol (4 mL/2 mL) in a 15 mL sealed pressure resistant reaction tube. An orange suspension was obtained after sonification. Then 0.5 mL of 3 M acetic acid was added. The pressure resistant glass reaction tube is subjected to the traditional three cycle method of liquid nitrogen freezing, degassing and thawing degassing. After that, place the sealed Pressure resistant reaction tube in an oven at 120 °C for 4 days. The precipitate was washed with 1,4-dioxane, anhydrous ethanol and THF successively. Extract the obtained powder with tetrahydrofuran Soxhlet for 12 hours to further remove the monomer molecules, and was dried at 120 °C under vacuum 12h. to give 51.6 mg of an orange powder in 84.6% yield. Elemental analysis revealed that the content of C, H, N and S to be 74.37%, 4.44%, 5.76% and 12.07%, which is quite close to the theoretical value of Py-TT-COF (C_30_H_17_N_2_S_2_, C 76.76%, H 3.62%, N 5.97%, S 13.65%)^[1]^.

**Py-BT COF powder:** 1,3,6,8-tetrakis(4-aminophenyl)pyrene (0.059 mmol, 32 mg) and 2,2'-bithiophene-5,5'-dicarboxaldehyde (0.112 mmol, 25 mg) were added to a mixture of mesitylene/benzyl alcohol (4 mL/2 mL) in a 15 mL sealed pressure resistant reaction tube. An orange suspension was obtained after sonification. Then 0.5 mL of 3 M acetic acid was added. The pressure resistant glass reaction tube is subjected to the traditional three cycle method of liquid nitrogen freezing, degassing and thawing degassing. After that, place the sealed pressure resistant reaction tube in an oven at 120 °C for 4 days. The precipitate was washed with 1,4-dioxane, anhydrous ethanol and THF successively, Extract the obtained powder with tetrahydrofuran Soxhlet for 12 hours to further remove the monomer molecules, and was dried at 120 °C under vacuum 12h to give 43.7 mg of an orange powder in 76.7% yield. Elemental analysis revealed that the content of C, H, N and S to be 75.94%, 4.51%, 5.15% and 11.02%, which is quite close to the theoretical value of Py-BT-COF (C_34_H_18_N_2_S_2_, C 78.74%, H 3.50%, N 5.40%, S 12.36%).

**3. Instrumentation and Methods**

The crystal structures were performed by X-ray diffraction (XRD) measurement (Bruker Rigaku SmartLab X-ray) using Cu Kα radiation source (λ=0.15418 nm). High-resolution transmission electron microscopy (TEM) images, inverse Fourier transform patterns were characterized by Thermo Fisher Scientific Themis 300. The surface chemical state was conducted through X-ray photoelectron spectroscopy (XPS) spectra (ESCALAB250XI). The C1s peak at 284 eV was used as a reference for the calibration. UV-Vis measurements were carried out on UV-Vis optical spectrophotometer (Perkin Elmer, Lambda 1050). The VERTEX 70v Fourier infrared spectrometer of BRUKER was employed to determine the structure and chemical bonds. The GIWAXS data were obtained at 1W1A Diffuse X-ray Scattering Station, Beijing Synchrotron Radiation Facility (BSRF-1W1A). The dielectric constant of COF films was measured using the ellipsometer UVISEL Plus from HORIBA France SAS, France.

**Transient Absorption Spectra (TAs):** At 370 nm excitation, the ultrafast transient absorption (TA) spectroscopy with femtosecond Ti: sapphire laser system (Coherent) and Helios pump-probe system (Ultrafast Systems) were measured. The laser was generated by Yb: KGW fiber laser system (PHAROS, Light Conversion, 1030 nm, 190 fs). The tunable optical parametric amplifiers can produce pump pulses which could be tuned from 350 nm-2600 nm. A small portion of fundamental beam is focused into a sapphire crystal to generate white light continuum, which is employed as probe. Measurements in the femtosecond (fs) range can be obtained by fixing a quartz substrate with grown COF nano-thin film onto the sample pool.

**Z-Scan Experiments:** In the Z-scan technology, an optical parametric amplifier (OPA, ORPHEUS, Light Conversion) were used to produce 190 fs pulse at selected wavelength and a Q-switched Nd:YAG laser (Surelite II, Continuum) were used to obtain 5 ns (FWHM) pulses of 532 nm. Measurements in the open-aperture Z-scan curves can be obtained by fixing a quartz substrate with grown COF nano-thin film onto the sample pool.

**Photocurrent density and Surface photovoltage spectra (SPV)：**The photocurrent density was measured using the CHI 760 electrochemical workstation from Shanghai Chenhua Technology Co., Ltd. Platinum wire served as the counter electrode, silver electrode as the reference electrode, and the working electrode consisted of a COF film on an ITO substrate (1×1 cm). The testing process involved using a 0.5 mol/L Na_2_SO_4_ solution as the electrolyte. The applied voltage was 0 V, and the sample was subjected to 20 S of irradiation from a 300 W xenon lamp. The surface photovoltage (SPV) tests were performed using the CEL-SPS1000 surface photovoltage testing system from Beijing Zhongjiao Jinyuan Technology Co., Ltd. The measurement parameters included a step size of 2 nm, a delay time of 1500 ms, and a measurement range of 250 nm to 650 nm.
**XPS:** The chemical composition and elemental valence state of Py-TT COF and Py-BT COF were characterized by XPS (Figure S5), shows the presence of carbon and nitrogen. The C1s XPS spectrum of Py-TT COF can be divided into several major peaks centered at around 284.7 eV, 285.9 eV, and 288.7 eV, which correspond to the, C–C/C=C, C=N, and π=π^*^, respectively. Similarly, the high-resolution XPS spectrum of N1 s is composed of two peaks which are centered at around 397.7 and 399.1 eV, which correspond to the, C=N, and unreacted amine groups (-NH_2_), respectively. The C1s XPS spectrum of Py-BT COF can be divided into several major peaks centered at around 284.7 eV, 285.9 eV, and 289.2 eV, which correspond to the, C–C/C=C, C=N, and π=π^*^, respectively^[2]^. Similarly, the high-resolution XPS spectrum of N1 s is composed of two peaks which are centered at around 397.7 and 399.1 eV, which correspond to the, C=N, and unreacted amine groups (-NH_2_), respectively. Meanwhile, a weak peak at around 288.7 and 289.2 eV is related to the interaction between the stacking layers of COF and chemical shift occurs in two different environments.

**BET:** The Permanent porosity of Py-TT COF and Py-BT COF were investigated using its nitrogen sorption isotherm at 77 K (Figure S6). Py-TT COF and Py-BT COF exhibited a reversible sorption with a typical type IV curve, which indicates the pores in Py-TT COF and Py-BT COF are mainly mesopores^[3]^. The specific surface areas of Py-TT COF and Py-BT COF are dependent on their microcrystalline structures, with values of 1145 m^2^/g and 1109 m^2^/g, respectively. Ordered, tetragonal pore structure of Py-TT COF and Py-BT-COF were clearly observed on high resolution transmission electron microscopy (HR-TEM) with a periodicity of ~1.9 and ~2.5 nm, which is consistent with the pore size proposed by the theoretical simulation of the BET surface area gives a value.

**CPD：**To describe the band structure, the work functions (WF) of the COFs films were determined using Kelvin probe force microscope (KPFM). The testing process was conducted in an argon environment to avoid the effects of humidity and oxygen adsorption on the material surface. The work function (WF_tip_) of the Pt probe is defined as 5.30 eV. By applying the formula^[4]^,

$${WF}_{sample}=e\times CPD+{WF}_{tip}$$

Where *e* represents the elementary charge, CPD denotes the contact potential difference between the sample and the probe, and WF_tip_ represents the work function of the probe. The work functions of Py-TT COF and Py-BT COF are determined to be 5.03 eV and 5.10 eV, respectively.


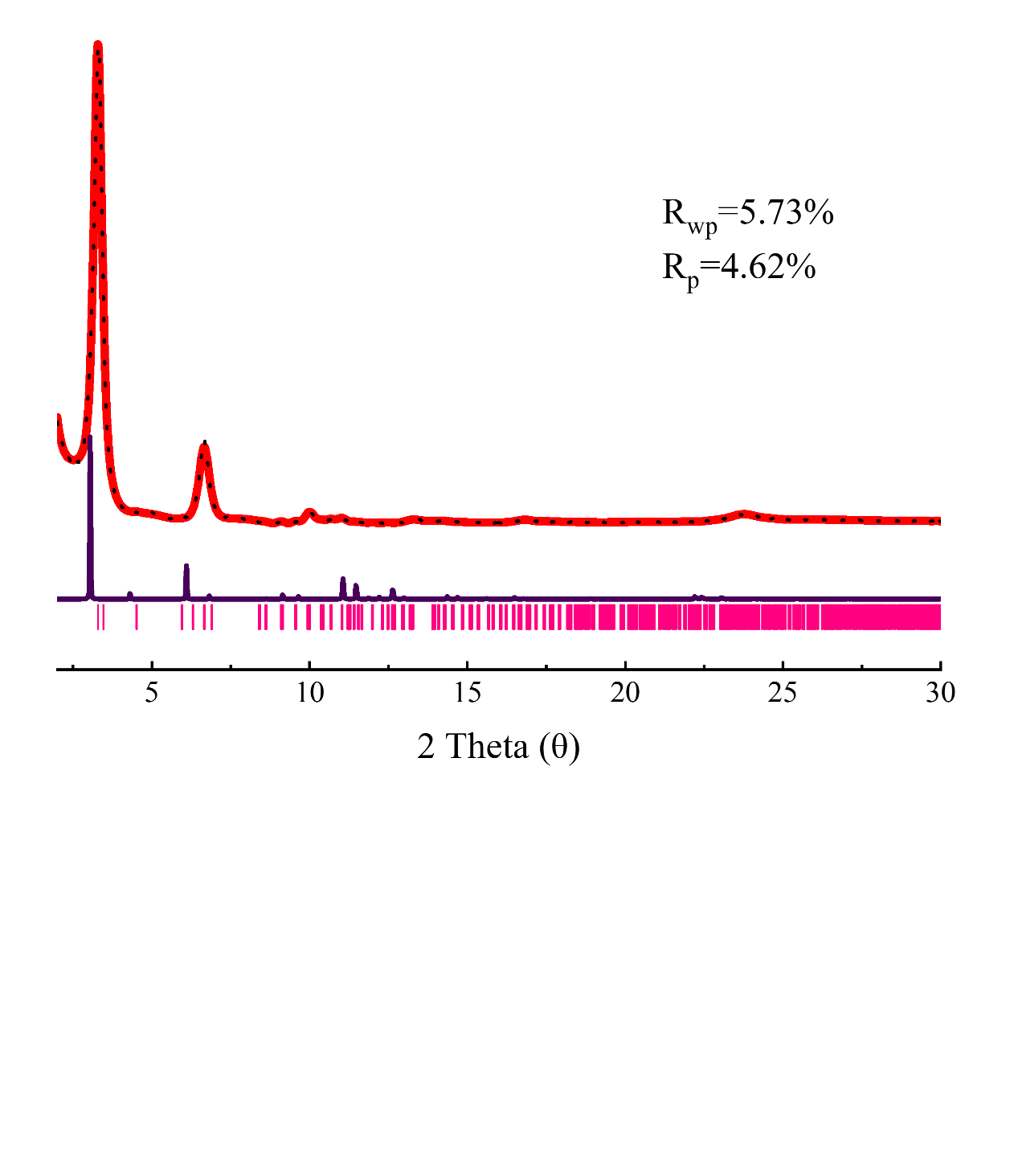


Figure S1. The XRD of Py-TT COF: simulated (brown), experimental (red), Pawley refined (black), and Bragg position (pink).


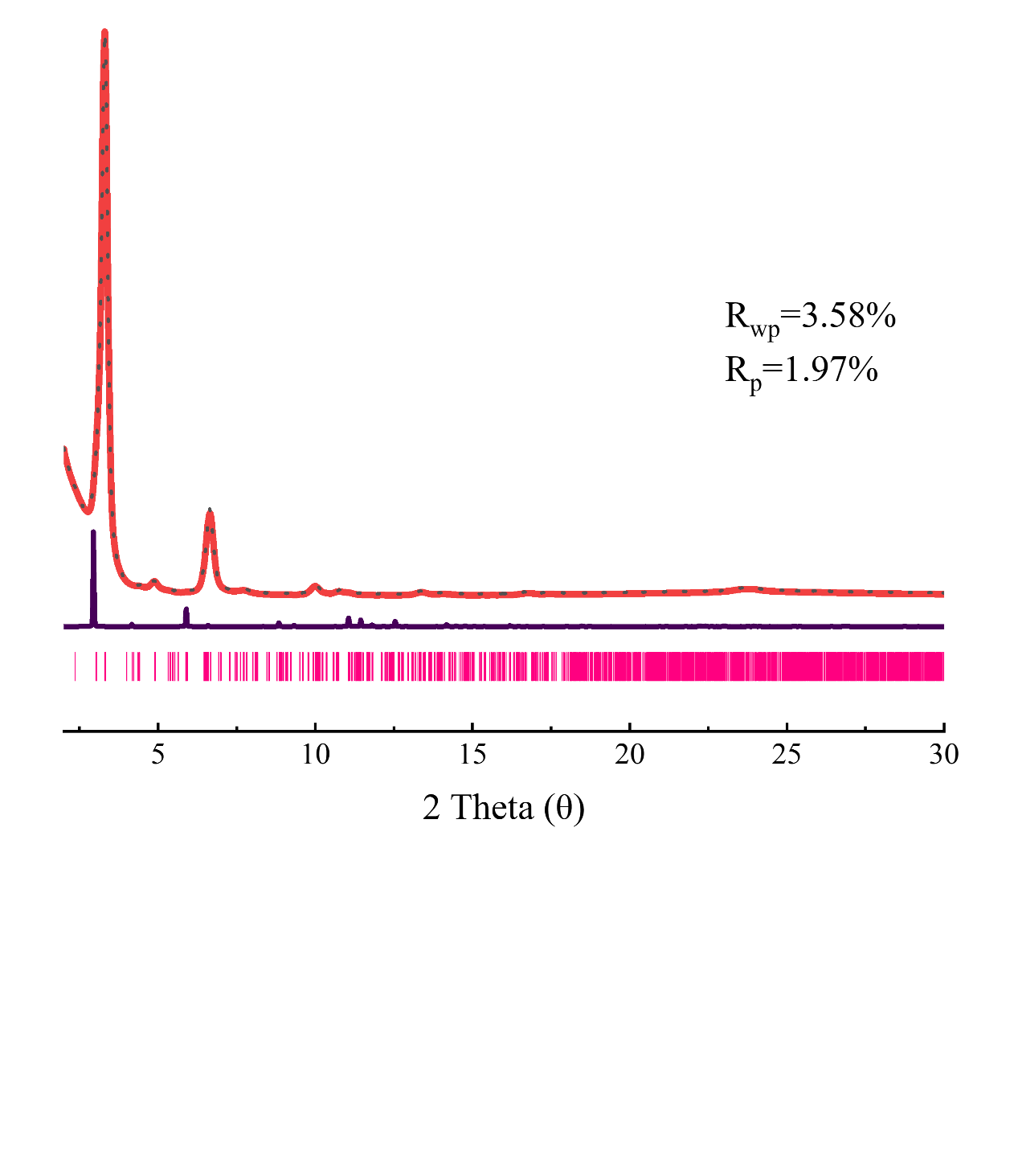


Figure S2. The XRD of Py-BT COF: simulated (brown), experimental (red), Pawley refined (black), and Bragg position (pink).


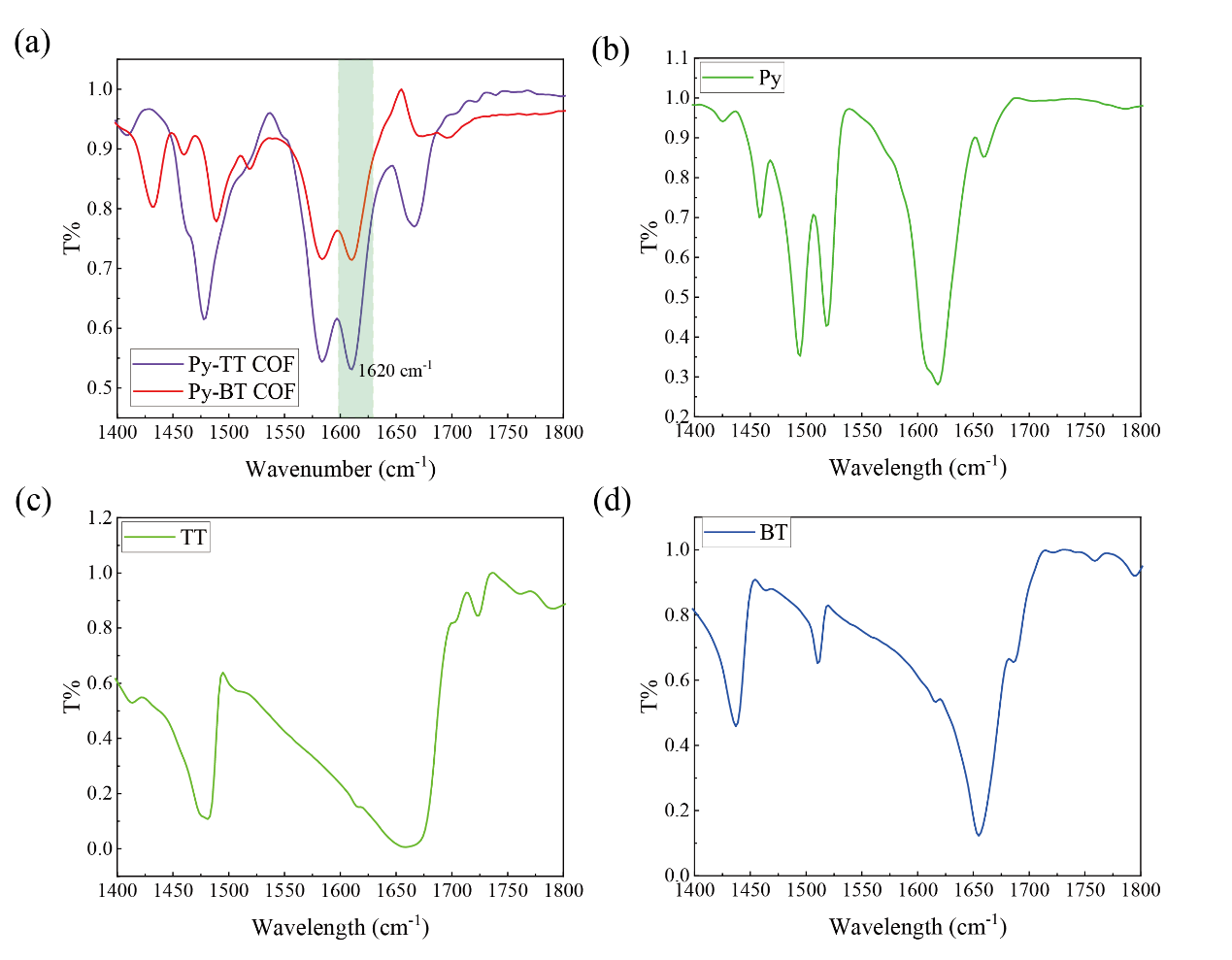


Figure S3. IR spectra comparison of Py-TT COF and Py-BT COF.


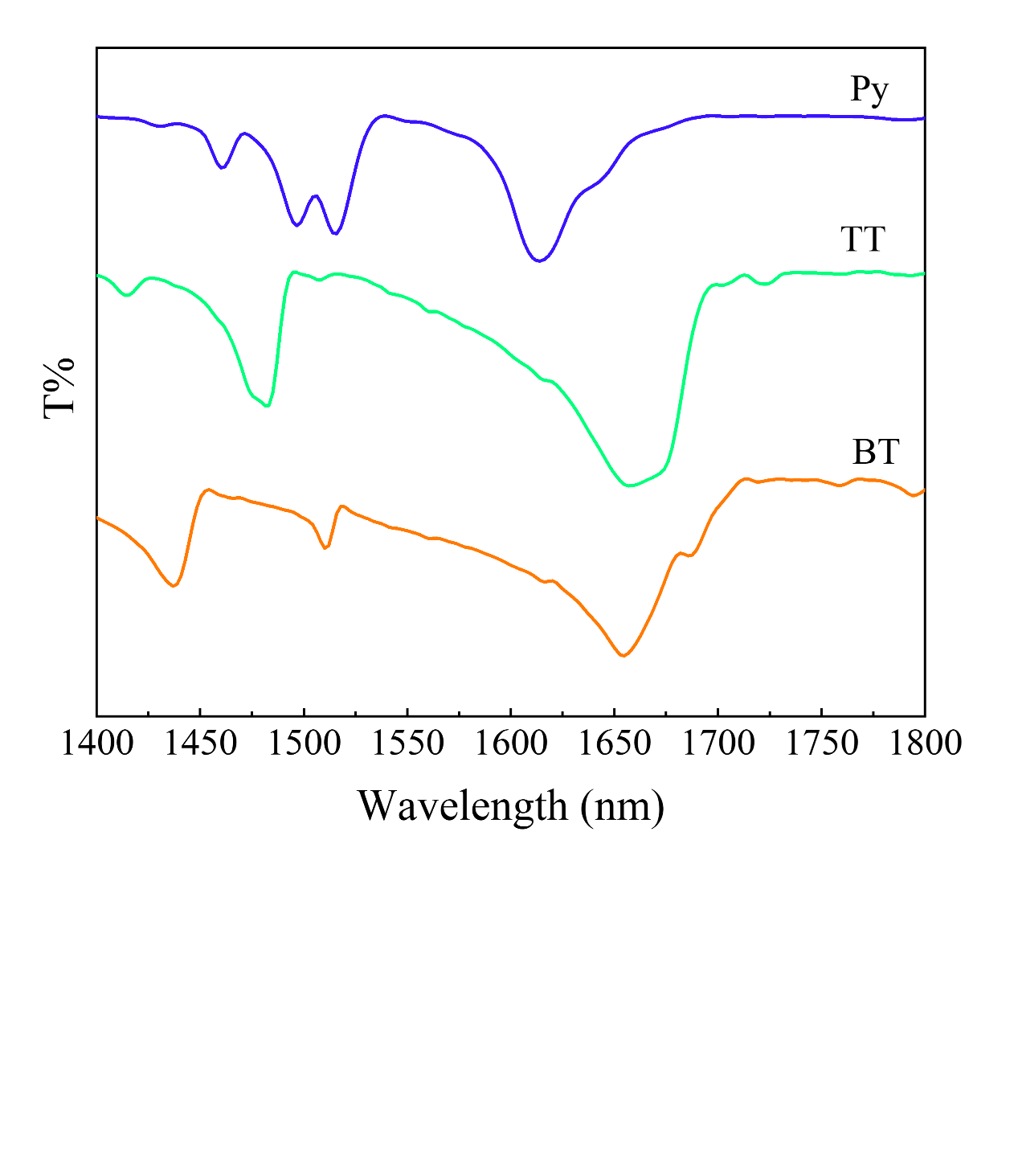


Figure S4. IR spectra comparison of TT, BT and Py.


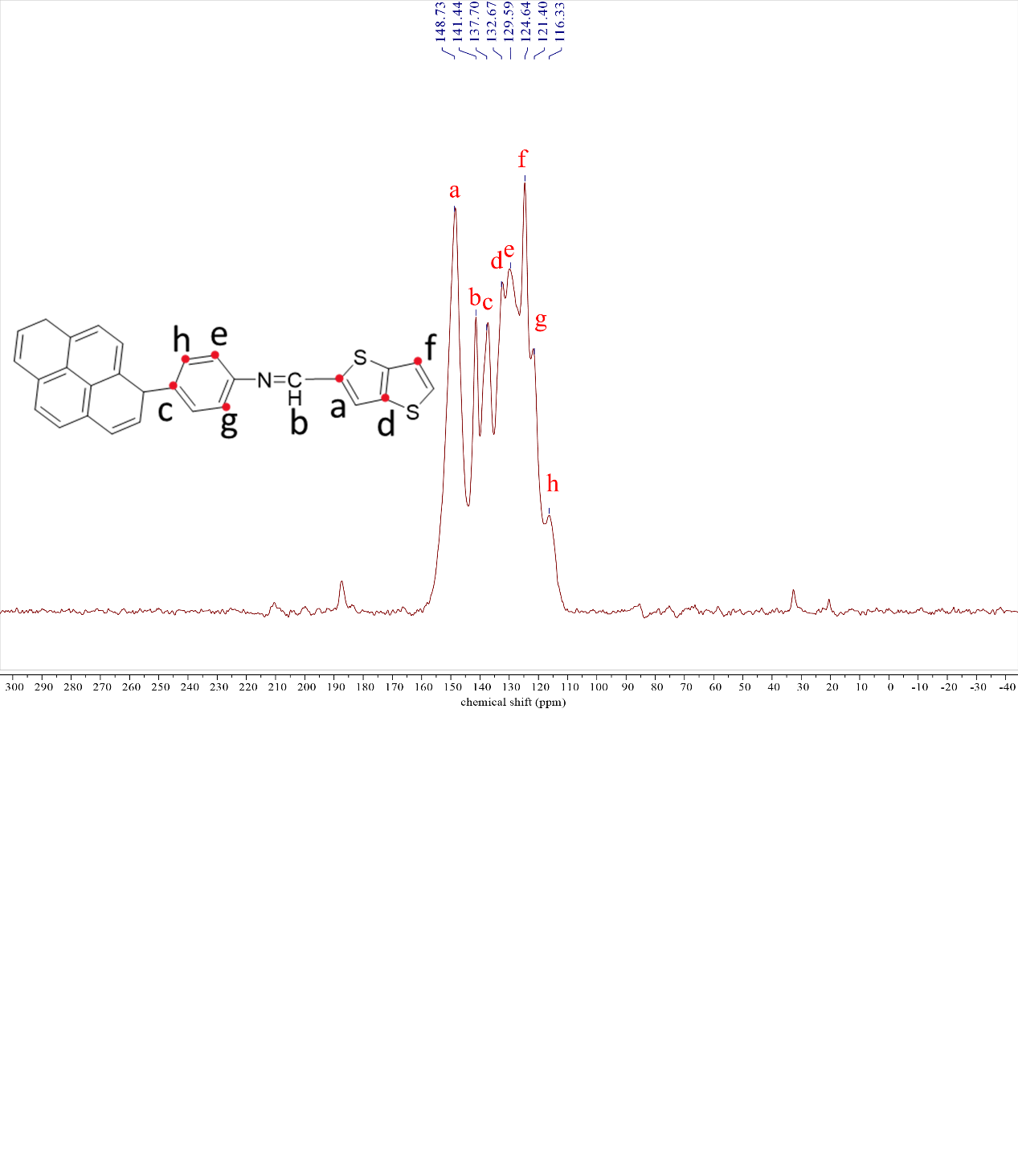


Figure S5. ^13^C CP-MAS NMR spectra of Py-TT COF.


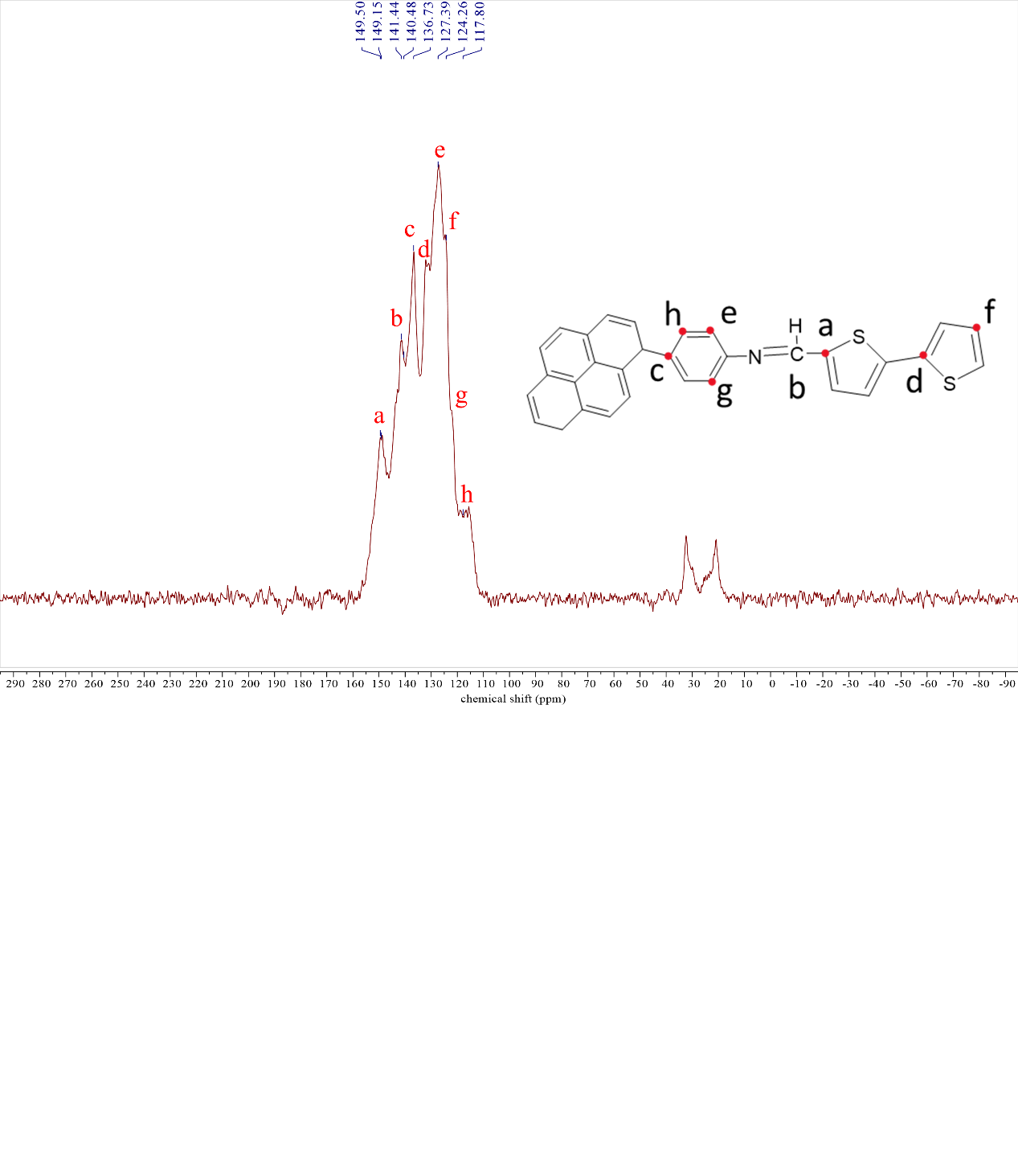


Figure S6. ^13^C CP-MAS NMR spectra of Py-BT COF.


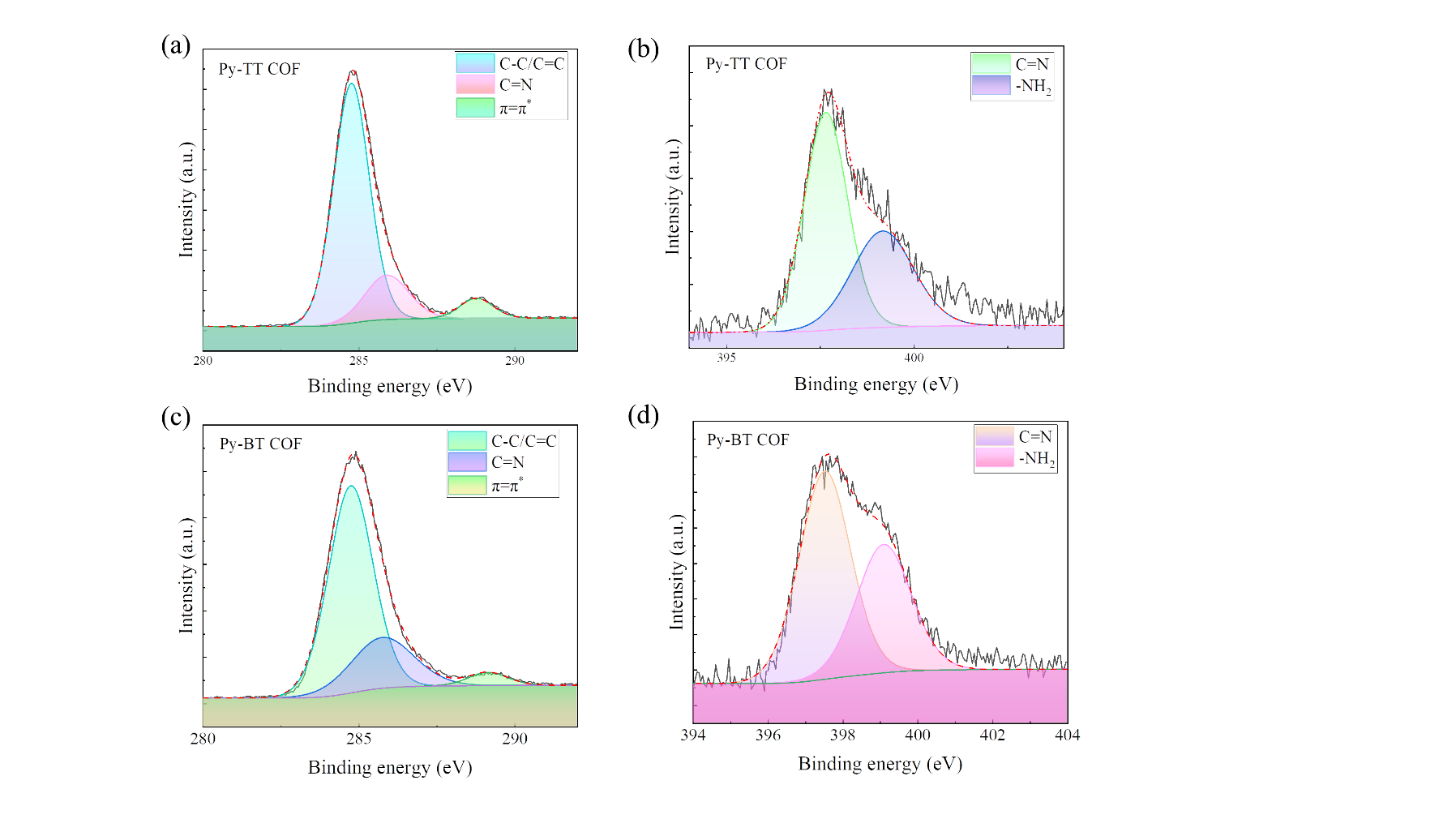


Figure S7. (a) C XPS spectra of Py-TT COF samples; (b) N XPS spectra of Py-TT COF samples; (c) C XPS spectra of Py-BT COF samples; (d) N XPS spectra of Py-BT COF samples.


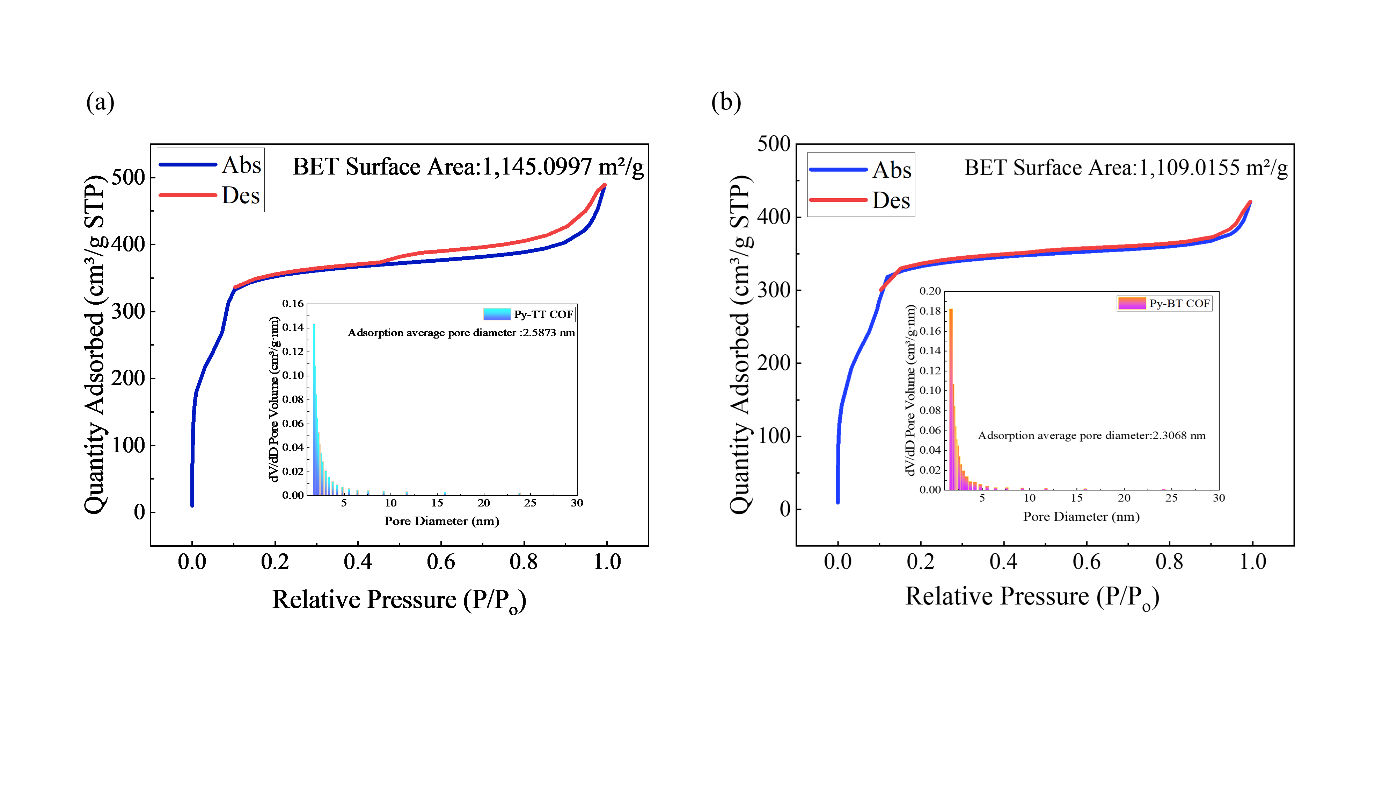


Figure S8. Specific surface area and pore size distribution of (a) Py-TT COF and (b) Py-BT COF.


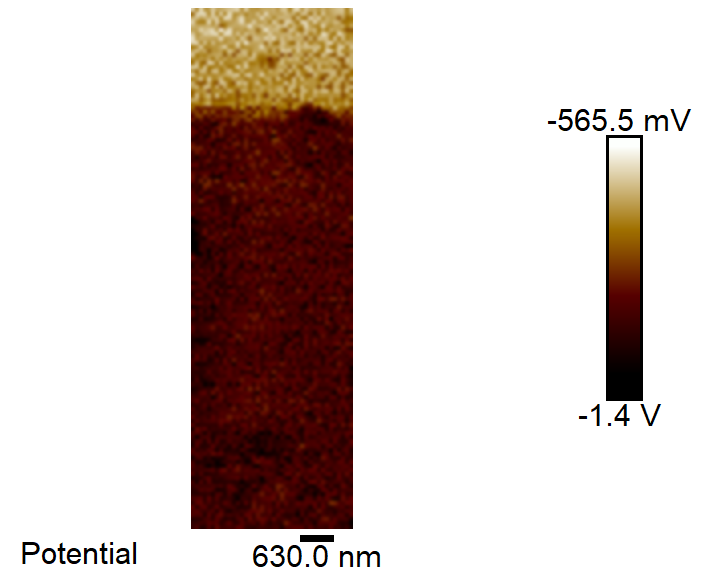


Figure S9. CPD values of Py-TT COF films tested by KPFM.


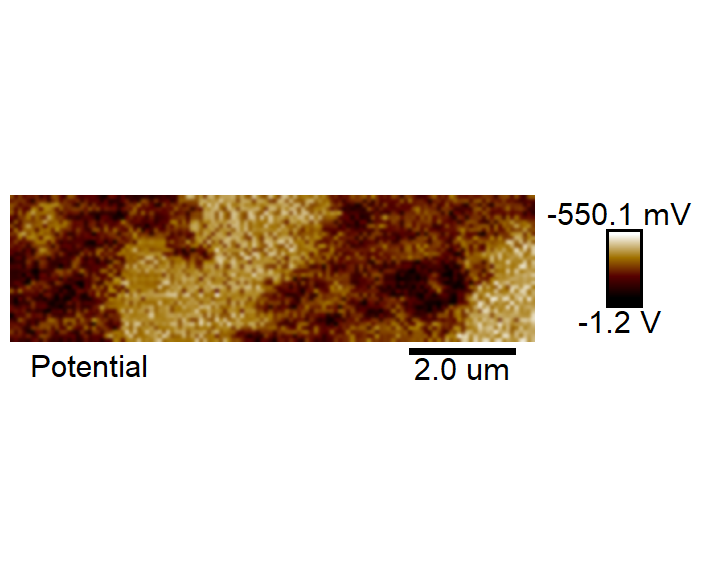


Figure S10 CPD values of Py-BT COF films tested by KPFM.


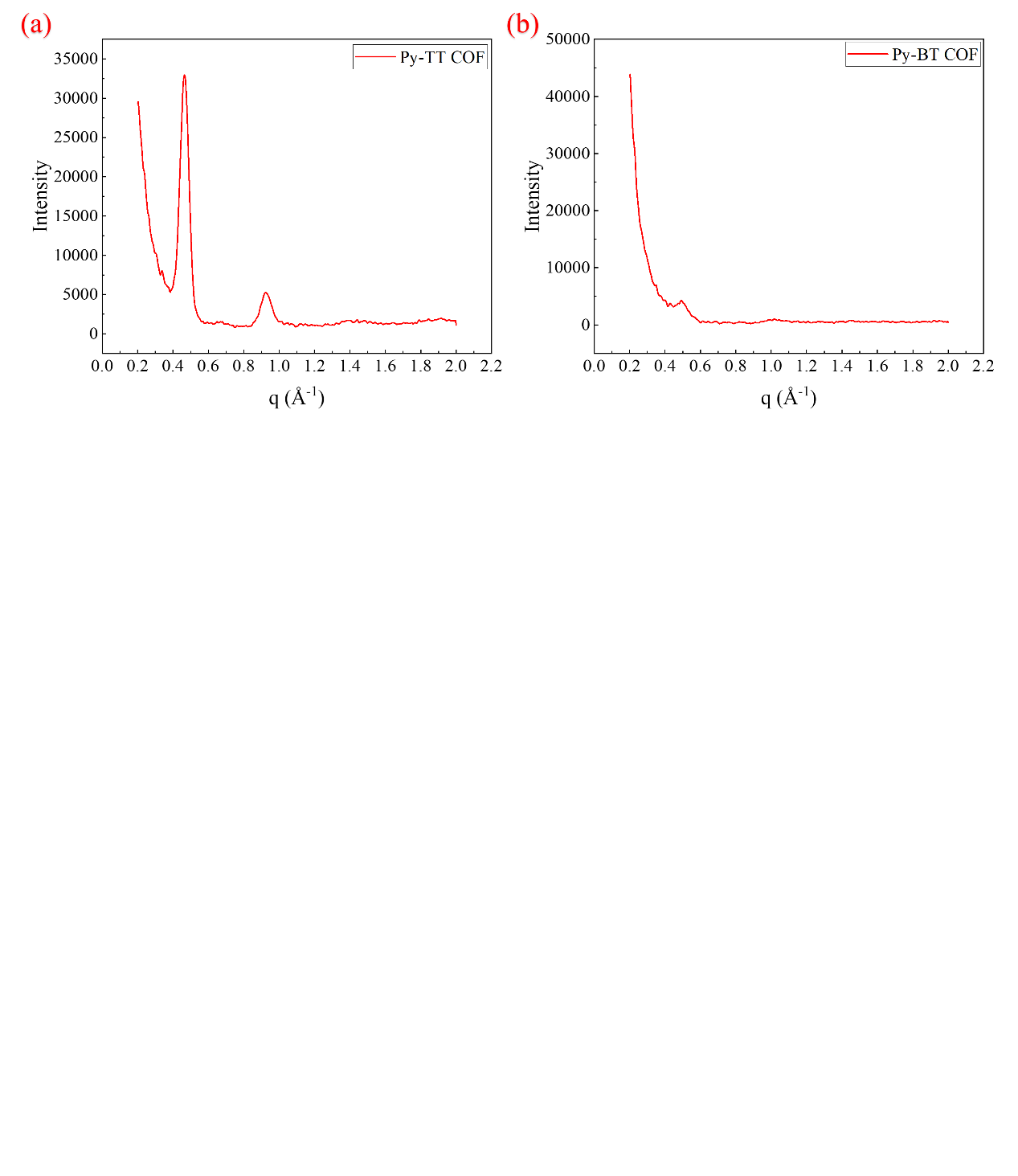


Figure S11. Line profile of (a) Py-TT COF film and (b)Py-BT COF film.


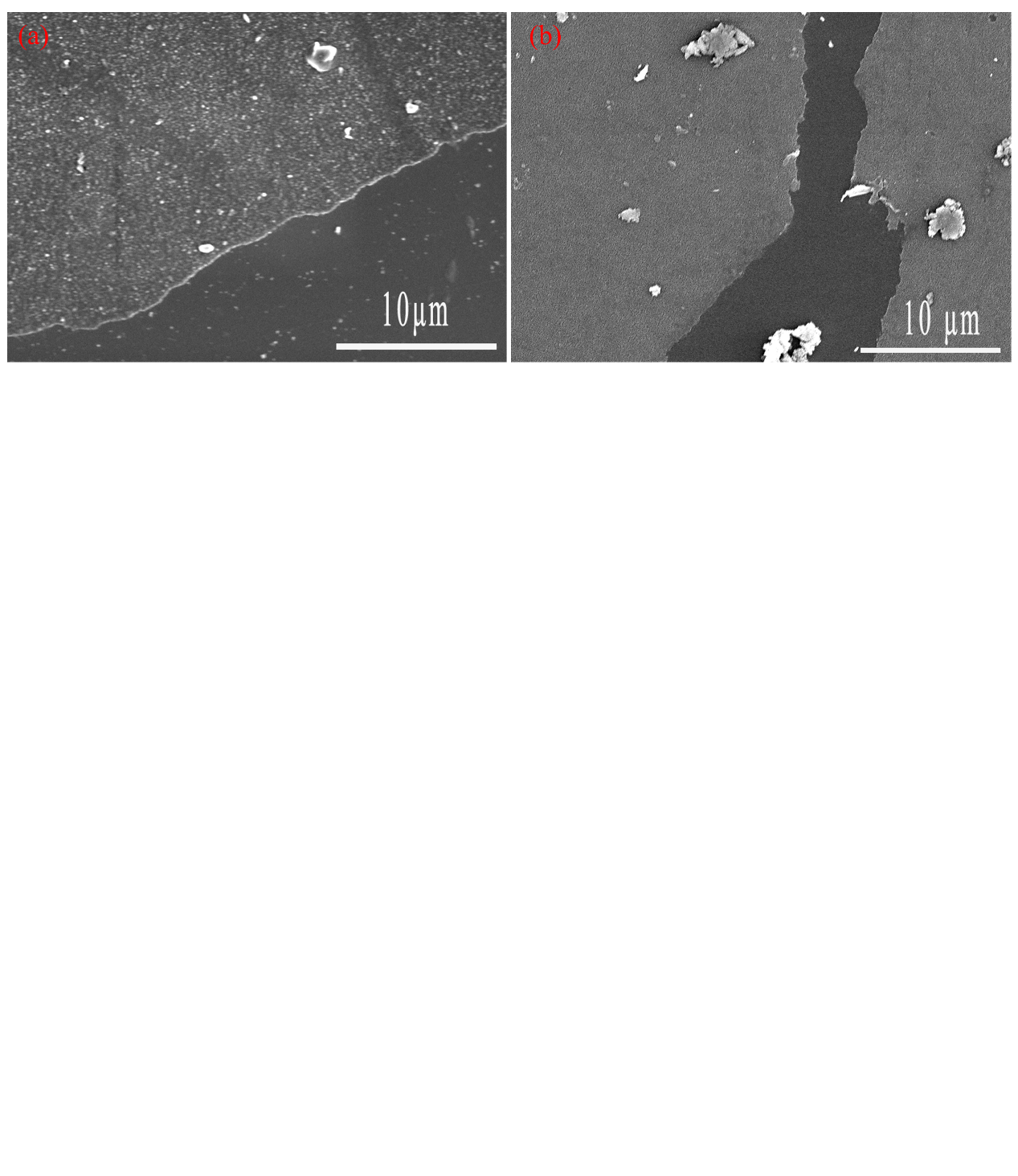


Figure S12. SEM images of the (a) Py-TT COF film and (b) Py-BT COF film.


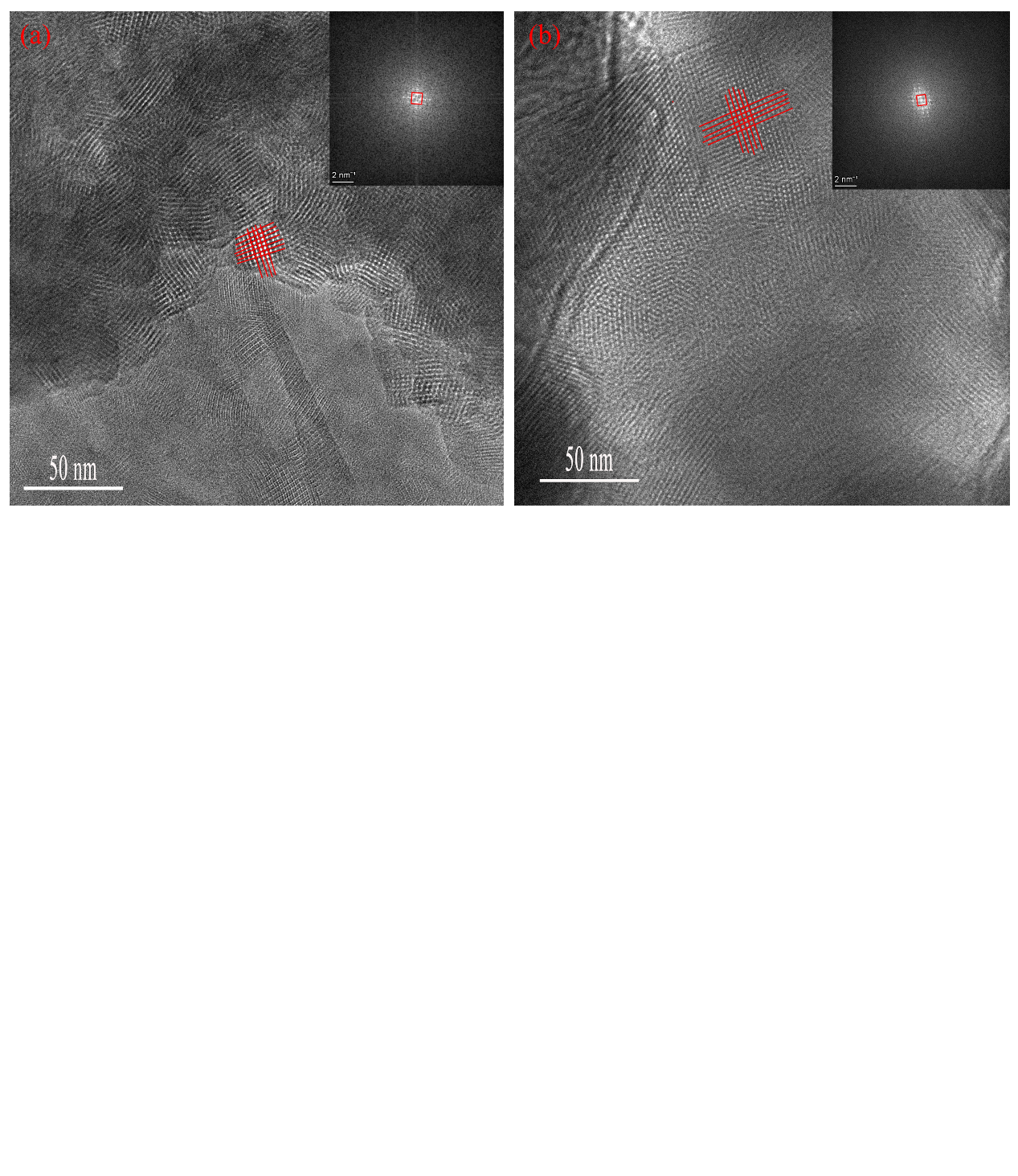


Figure S13. HR-TEM and Inverse Fourier transform electron diffraction maps images of the (a) Py-TT COF film and (b) Py-BT COF film.


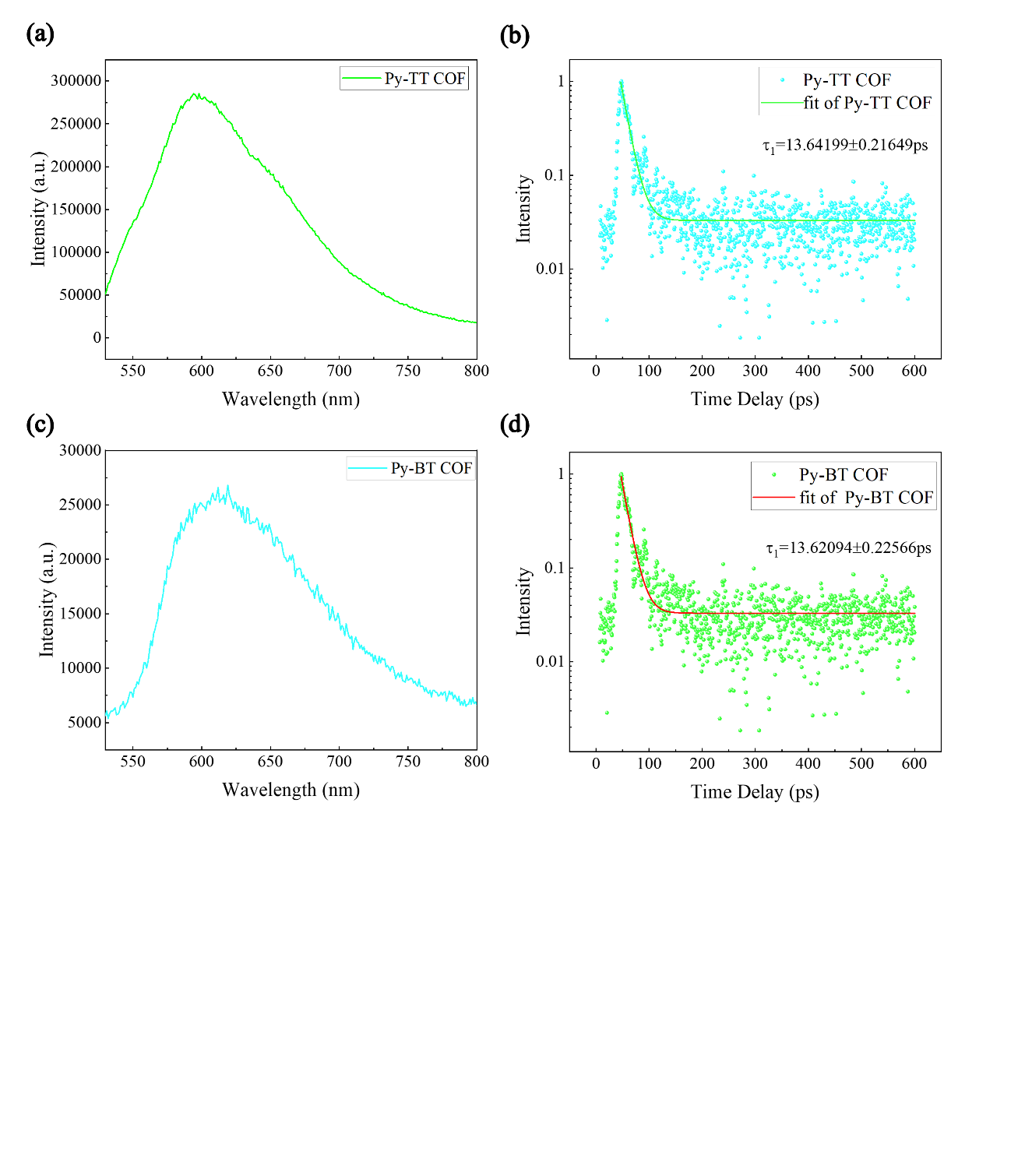


Figure S14. Py-TT COF (a) fluorescence spectrum and (b) ultrafast fluorescence lifetime; Py-BT COF (c) fluorescence spectrum and (d) ultrafast fluorescence lifetime.


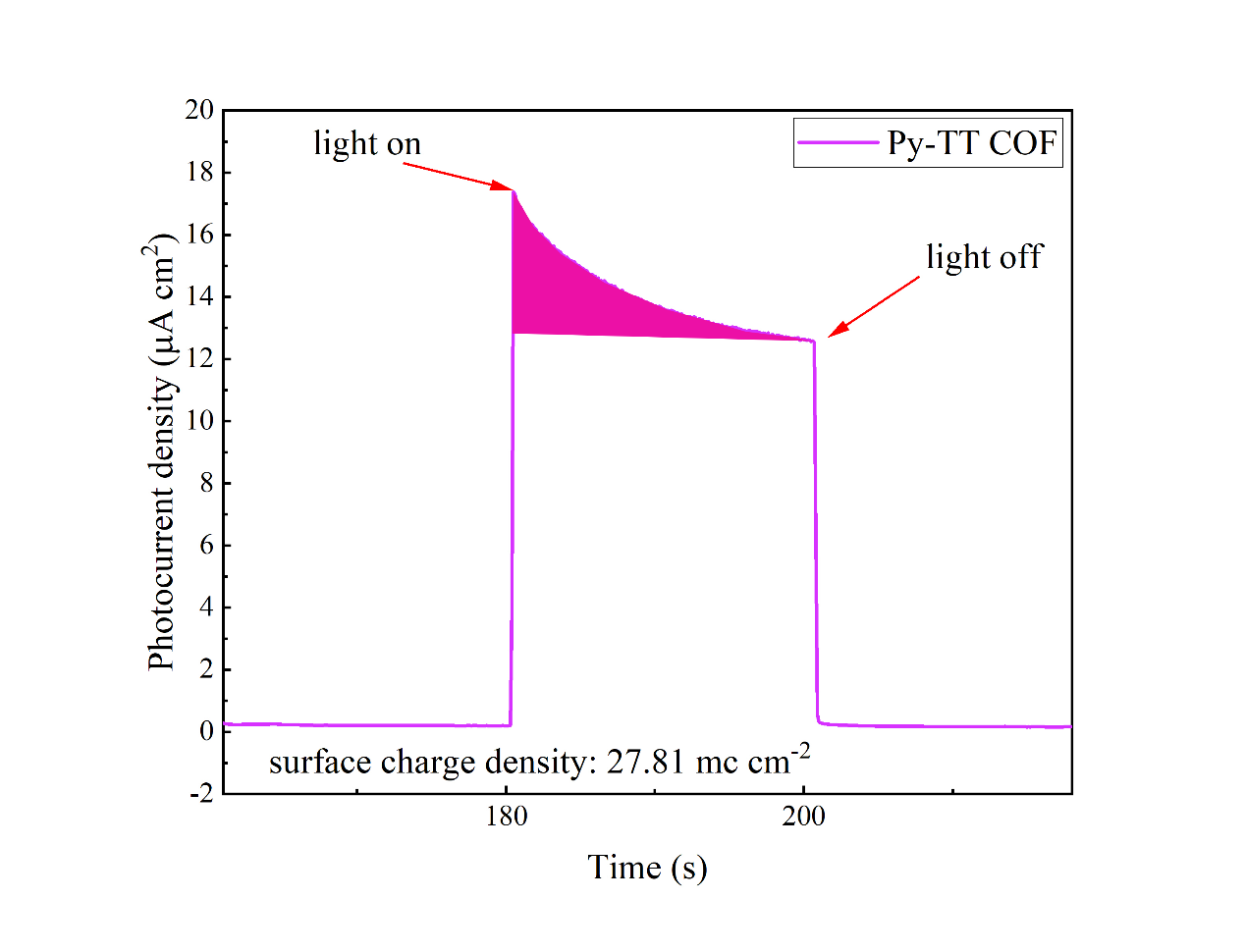


Figure S15. The transient photocurrent density of Py-TT COF sample.


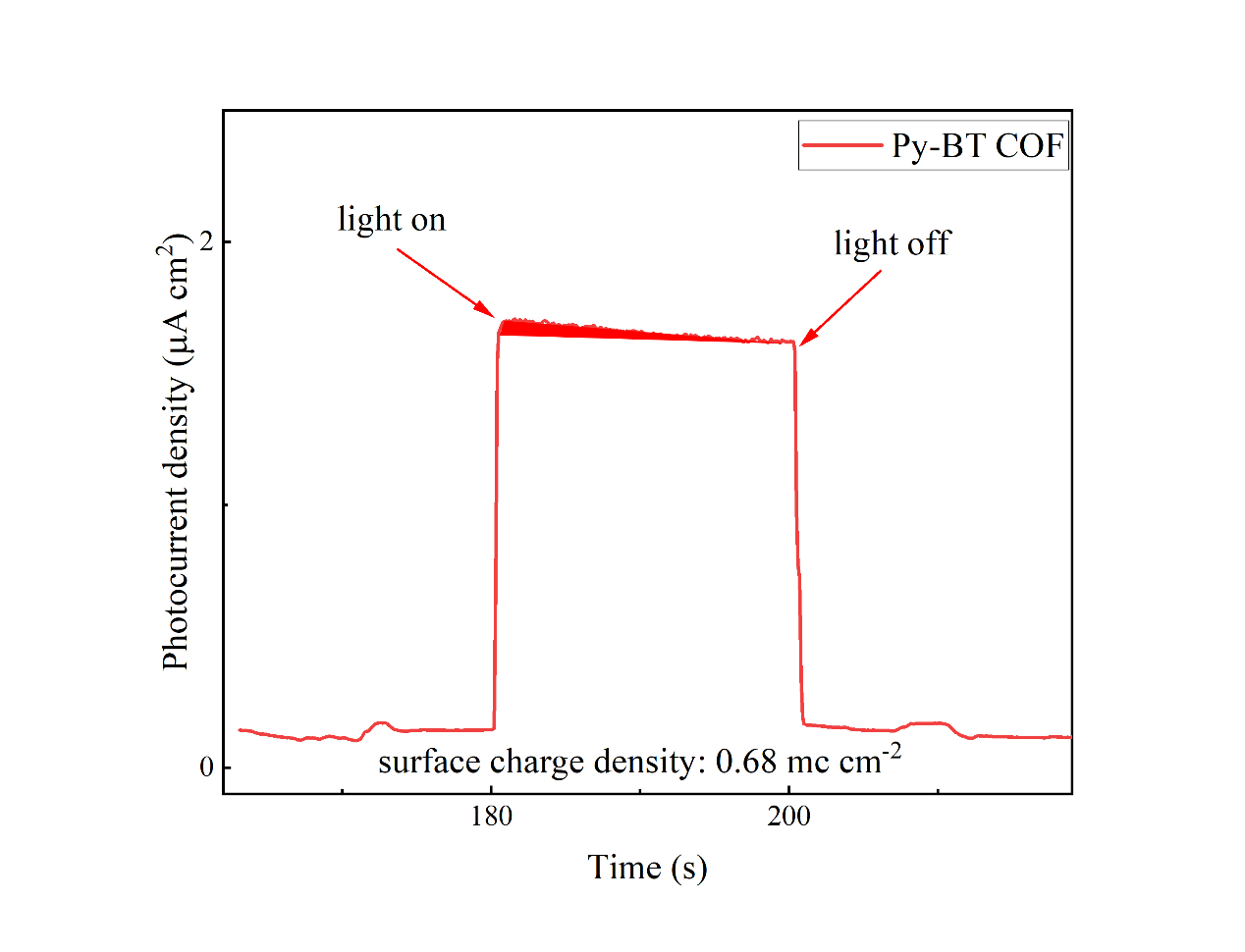


Figure S16. The transient photocurrent density of Py-BT COF sample.


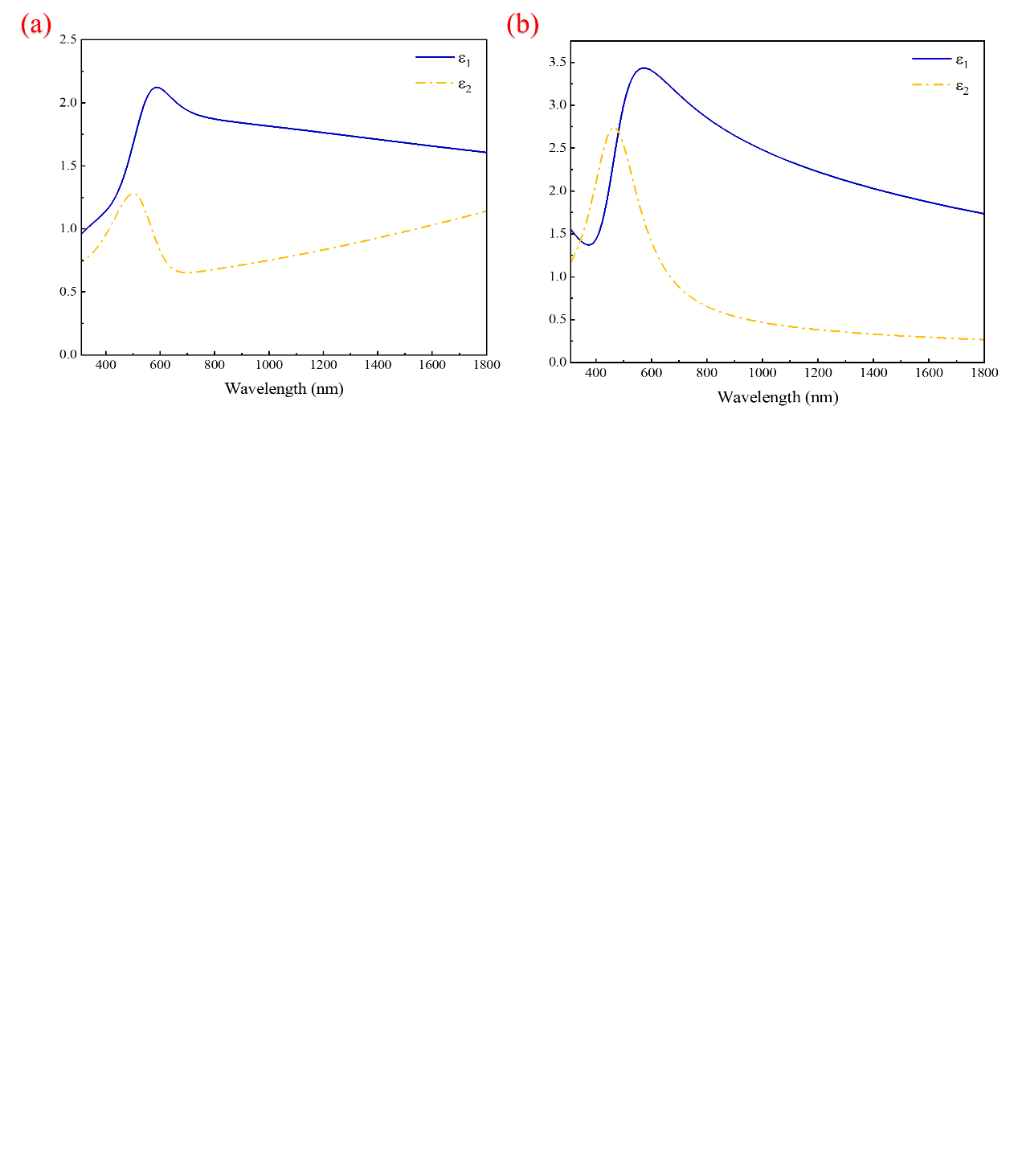


Figure S17. Real (solid curve) and imaginary parts (dashed curve) of the refractive index of (a) Py-TT COF nanofilms and (b) Py-BT COF nanofilms.


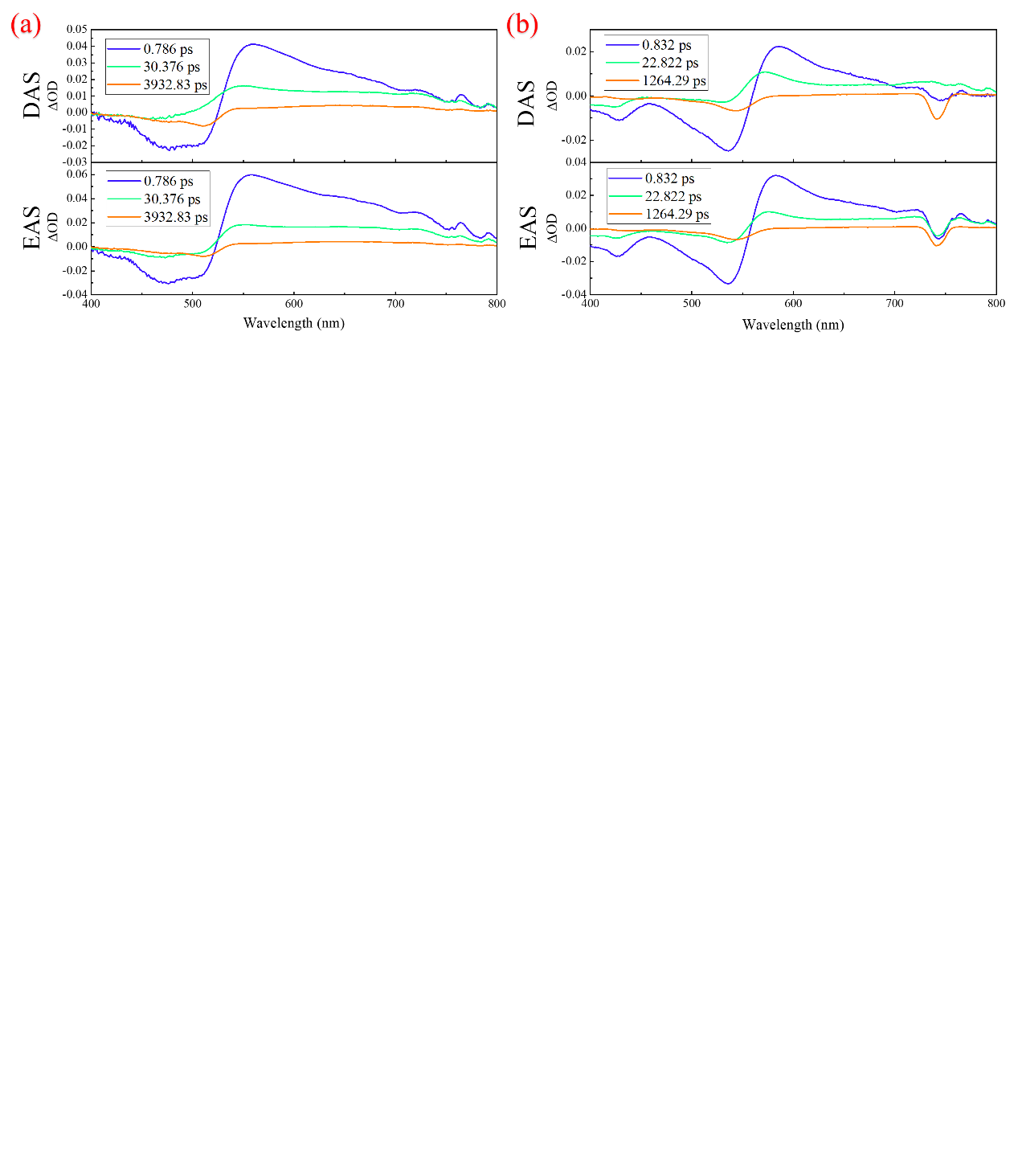


Figure S18. EAS and DAS spectra of (a) Py-TT COF nanofilm and (b) Py-BT COF nanofilm.

[1] S. Li, Y. Z. Liu, L. Li, C. X. Liu, J. N. Li, S. Ashraf, P. F. Li, B. Wang, *Acs Applied Materials & Interfaces* **2020**, *12*, 22910-22916.

[2] a M. H. Liu, Y. X. Liu, J. C. Dong, Y. C. Bai, W. Q. Gao, S. C. Shang, X. Y. Wang, J. H. Kuang, C. S. Du, Y. Zou, J. Y. Chen, Y. Q. Liu, *Nature Communications* **2022**, *13*, 1411; b B. C. Luo, Y. Chen, Y. B. Zhang, J. Q. Huo, *Journal of Catalysis* **2021**, *402*, 52-60; c H. Zhao, H. Chen, C. Y. Xu, Z. H. Li, B. Ding, H. Dou, X. G. Zhang, *Acs Applied Energy Materials* **2021**, *4*, 11377-11385.

[3] A. Nagai, X. Chen, X. Feng, X. S. Ding, Z. Q. Guo, D. L. Jiang, *Angewandte Chemie-International Edition* **2013**, *52*, 3770-3774.

[4] a J. Yang, J. Jing, W. Li, Y. Zhu, *Advanced Science* **2022**, *9*, 2201134; b D. m. Zhao, Y. q. Wang, C. L. Dong, Y. C. Huang, J. Chen, F. Xue, S. Shen, L. Guo, *Nature Energy* **2021**, *6*, 388-397.
